# Potent Virustatic Polymer–Lipid Nanomimics Block Viral Entry and Inhibit Malaria Parasites In Vivo

**DOI:** 10.1021/acscentsci.1c01368

**Published:** 2022-05-03

**Authors:** Adrian Najer, Joshua Blight, Catherine B. Ducker, Matteo Gasbarri, Jonathan C. Brown, Junyi Che, Håkon Høgset, Catherine Saunders, Miina Ojansivu, Zixuan Lu, Yiyang Lin, Jonathan Yeow, Omar Rifaie-Graham, Michael Potter, Renée Tonkin, Jelle Penders, James J. Doutch, Athina Georgiadou, Hanna M. G. Barriga, Margaret N. Holme, Aubrey J. Cunnington, Laurence Bugeon, Margaret J. Dallman, Wendy S. Barclay, Francesco Stellacci, Jake Baum, Molly M. Stevens

**Affiliations:** † Department of Materials, Department of Bioengineering, and Institute of Biomedical Engineering, Imperial College London, London, SW7 2AZ, U.K.; ‡ Department of Life Sciences, Imperial College London, London, SW7 2AZ, U.K.; § Institute of Materials, Ecole Polytechnique Fédérale de Lausanne (EPFL), 1015 Lausanne, Switzerland; ∥ Department of Infectious Disease, Imperial College London, London, W2 1PG, U.K.; ⊥ Department of Medical Biochemistry and Biophysics, Karolinska Institutet, SE-171 77 Stockholm, Sweden; # Rutherford Appleton Laboratory, ISIS Neutron and Muon Source, STFC, Didcot OX11 ODE, U.K.; ∇ Institute of Bioengineering, Ecole Polytechnique Fédérale de Lausanne (EPFL), 1015 Lausanne, Switzerland

## Abstract

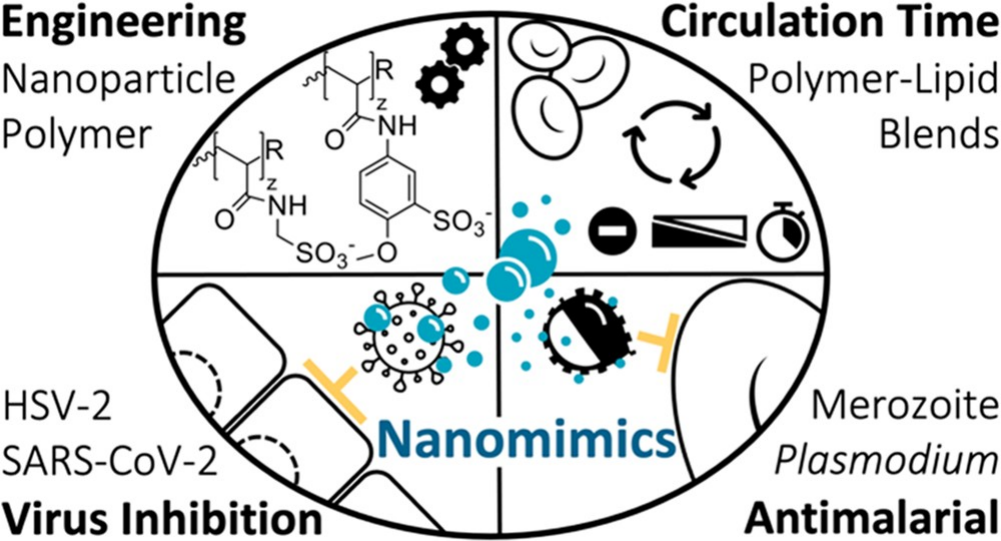

Infectious diseases continue to pose a substantial burden on global populations, requiring innovative broad-spectrum prophylactic and treatment alternatives. Here, we have designed modular synthetic polymer nanoparticles that mimic functional components of host cell membranes, yielding multivalent nanomimics that act by directly binding to varied pathogens. Nanomimic blood circulation time was prolonged by reformulating polymer–lipid hybrids. Femtomolar concentrations of the polymer nanomimics were sufficient to inhibit herpes simplex virus type 2 (HSV-2) entry into epithelial cells, while higher doses were needed against severe acute respiratory syndrome coronavirus 2 (SARS-CoV-2). Given their observed virustatic mode of action, the nanomimics were also tested with malaria parasite blood-stage merozoites, which lose their invasive capacity after a few minutes. Efficient inhibition of merozoite invasion of red blood cells was demonstrated both *in vitro* and *in vivo* using a preclinical rodent malaria model. We envision these nanomimics forming an adaptable platform for developing pathogen entry inhibitors and as immunomodulators, wherein nanomimic-inhibited pathogens can be secondarily targeted to sites of immune recognition.

## Abstract 2

Many viruses, including HSV-2 and SARS-CoV-2, and malaria parasites use the same host cell receptors for initial interaction. We designed host-mimicking nanoparticles to inhibit varied pathogens.

## Introduction

Infectious diseases constitute an immense health and economic burden on global health. This burden of disease is not limited to threats from endemic or emergent viral infections, such as the severe acute respiratory syndrome coronavirus 2 (SARS-CoV-2) responsible for the COVID-19 pandemic. Parasitic diseases such as malaria—one of the oldest and most prevalent infectious diseases—constitute an additional tremendous health burden on many of the poorest nations. Indeed, half of the world’s population is at risk of contracting malaria caused by various *Plasmodium* species, which in 2020 infected 241 million people globally and killed 627 000 people, most of whom were children in sub-Saharan Africa.^1^ Given the still unmet challenge of developing a broadly efficacious vaccine against complex protozoan pathogens like malaria, combined with the unpredictability of emergent new viruses, there is a strong case for developing easily deployable, alternative broad-spectrum prophylactic and treatment strategies. Such novel broad-spectrum prophylactic interventions may be particularly important when efficacious treatments are absent as in the early stages of a pandemic or in the case where vaccines remain elusive.

Materials technology, including nanotechnology, provides a relatively untapped route to designing broad-spectrum strategies against intracellular pathogens such as viruses or parasites. Nanoparticle-based pathogen inhibitors designed to date have principally used or mimicked host cell receptors, such as sialic acid or heparan sulfate, which are negatively charged residues used by many pathogens to bind and successfully enter host cells.^2,3^ Such synthetic inhibitors function by occupying pathogen ligands and therefore disturbing host cell entry.

Herpes simplex virus type 2 (HSV-2) is a common model virus that has been demonstrated to be susceptible to inhibition by various inhibitors ranging from heparin and multivalent gold nanoparticles (AuNPs),^4^ to dendrimers,^5^ and nanogels.^6^ Similarly, polyanionic structures,^7−9^ nanosponges,^10^ and nanodecoys^11^ have been shown to have inhibitory activity against SARS-CoV-2. Challenges for anionic inhibitors, intended for *in vivo* applications, are, however, their typically low potency, potentially unwanted anticoagulation activity, and rapid dilution plus elimination upon administration, as for example found when trying to translate polyanionic inhibitors for human immunodeficiency virus (HIV) infections.^12^ In contrast to nanomedicine-based viral inhibition, nanotechnological strategies against malaria to date have mainly been leveraged to provide antimalarial drug delivery and vaccine vehicles.^13−15^ Direct inhibition of parasite (merozoite) host cell entry via multivalent nanoparticle interactions is a little-explored avenue that could yet provide new treatment alternatives to conventional drugs. Most efforts with respect to merozoite invasion inhibition have focused on soluble heparin-like polysaccharides.^16−19^ Among the few nanoparticle studies, liposomes,^20^ polymersomes,^21^ and inorganic nanoparticles^22^ have been trialed for inhibition of merozoite invasion *in vitro*. However, these antiparasitic nanoscale inhibitors were all heparin based and have struggled to meet the challenges of achieving high efficacy, *in vivo* applicability, and extended blood circulation half-lives.^20^

Here, we present the design and testing of modular synthetic polymer and polymer–lipid nanomimics that achieve potent virus and parasite entry inhibition. We demonstrate formulation of cytocompatible and serum-stable nanoparticles presenting different types of sulfonated polymers on the particle surface. Further, coassembly of the copolymer with lipids, including a poly(ethylene glycol)-modified lipid (PEG-lipid), enabled fine-tuning of the nanoparticle surface charge to extend blood circulation times, as measured in a zebrafish embryo model. Testing of our polymer nanomimics revealed successful host cell entry inhibition of both HSV-2 and SARS-CoV-2 through a virustatic inhibition mechanism. Similarly, *in vitro* tests demonstrated potent malaria parasite invasion inhibitory activities of nanomimics across several *Plasmodium* parasite species. *In vivo* testing confirmed this activity in a rodent malaria model. Combined, these data demonstrate the versatility of our nanomimics as potent virus and parasite inhibitors that could yield urgently needed alternative treatment and prophylactic strategies against infectious pathogens.

## Results and Discussion

### Precise Surface Engineering of Biocompatible and Serum-Stable Polymer Nanomimics

**Figure 1:**
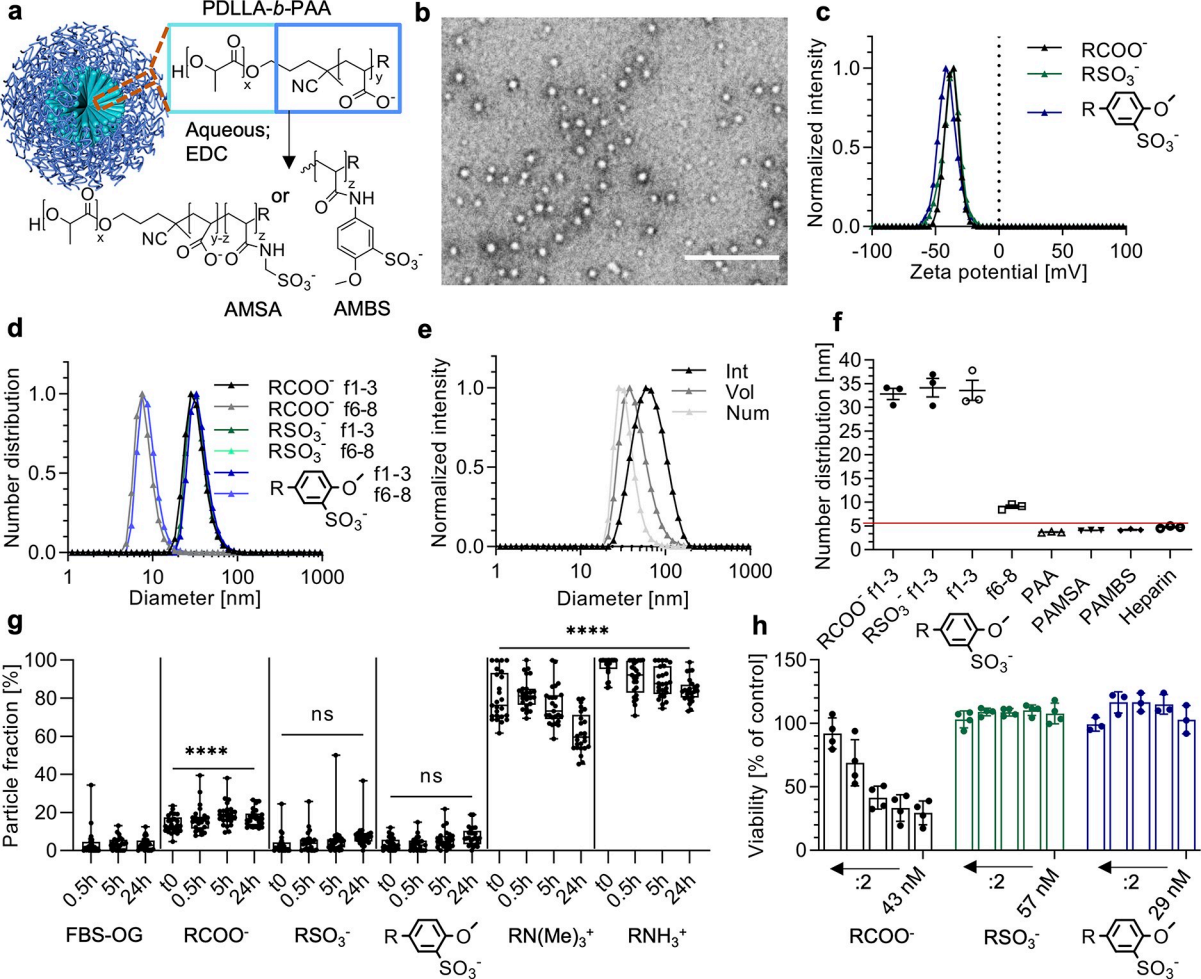
Polymer nanomimic design and characterization. (a) Schematic representation of PDLLA-*b*-PAA nanoparticle modification with aminomethanesulfonic acid (AMSA) and 5-amino-2-methoxybenzenesulfonic acid (AMBS). Nanoparticle schematic reproduced from ref (26) with permission. Copyright 2016 the Royal Society of Chemistry. (b) TEM image of polymer nanomimics. Scale bar, 200 nm. (c, d) Average zeta potential and DLS number distribution of various nanomimics, f1–3 and f6–8, correspond to the SEC fractions (technical triplicates). (e) Average DLS size distribution (intensity, volume, and number distribution) of sulfonated (AMSA) nanomimics (technical triplicates). (f) Average DLS number distribution (mean ± s.e.m., technical triplicates) for nanoparticle samples versus the hydrophilic polymer only (10 kDa PAA, and corresponding modified polymers PAMSA and PAMBS, and heparin 18 kDa; red line indicates kidney filtration size cutoff^27^). (g) Nonspecific binding of FBS-OG488 to various nanoparticles over time as obtained by two-component FCS fits (*n* = 25 technical replicates, one-way ANOVA with Tukey’s multiple comparisons test, shown comparisons to FBS-OG488 only, ****P < 0.0001, ns = not significant). (h) Cytocompatibility of nanomimics tested with the RAW 264.7 cell line compared to PBS controls (mean ± s.d., *N* ≥ 3 independent experiments with technical triplicates). Highest particle concentrations are given, while the subsequent values correspond to a two-fold serial dilution. Box-plots: center line, the median; box limits, upper and lower quartiles; whiskers, minimum and maximum values.

Biocompatibility, degradability, and a simple nanoparticle design, while keeping scalability and cost in mind, are key considerations when formulating nanomedicines for clinical translation. To build our polymer nanomimics, we utilized a simple amphiphilic block copolymer structure. The copolymer we decided to employ for this purpose is poly(dl-lactide)-*block*-poly(acrylic acid) (PDLLA-*b*-PAA, 9 kDa-9 kDa).^23,24^ This copolymer provided the necessary repetitive pathogen-binding units (hydrophilic PAA), which can be easily chemically modified to adjust the surface chemistry in a simple, modular fashion, connected to a degradable hydrophobic block (PDLLA). This allows optimization of pathogen binding by mimicking some properties of the host cell membranes, namely, heparan sulfate receptors, on the nanoparticle surface to yield nanomimics (Figure 1a).

Aqueous self-assembly through a solvent injection method or direct bulk hydration of the copolymer produced spherical nanoparticles with diameters of 19 ± 9 nm (*n* = 461) when measured in the dry state by transmission electron microscopy (TEM, Figure 1b, Supplementary Figure 1). Similar assembly of related PLLA–*b*-PAA (4.5 kD-18 kDa) formed cylindrical nanoparticles (Supplementary Figure 1). Size exclusion chromatography (SEC) was used to separate the main particle fraction used herein (f1–3), with a hydrodynamic diameter of around 30–50 nm when measured by dynamic light scattering (DLS), from smaller particles (f6–8, Figure 1d–f). Importantly, the initial nanoparticle sizes are above the renal cutoff size of about 5.5 nm,^25^ while the hydrophilic blocks alone are below that cutoff (Figure 1f, red line).

By employing the versatility of purely synthetic constructs, we subsequently tuned the pathogen-binding polymers (PAA) through direct surface modification on the assembled nanoparticles to obtain nanomimics that present heparan sulfate mimicking polymers on the surface. We installed various sulfonate moieties with increasing spacer lengths using aminomethanesulfonic acid (AMSA), 5-amino-2-methoxybenzenesulfonic acid (AMBS), and other molecules, keeping the anionic charge of the original PAA-based nanoparticle consistent (Figure 1c, Supplementary Figure 2). The anionic polymer concentration and change in the nature of the anionic charge could be followed using modified Farndale^28^ and toluidine blue (TB) microassays (Supplementary Figure 3). By coincorporation of cationic moieties at varying concentrations, precise adjustment of the zeta potential of the nanoparticles from negative to neutral to positive was achieved (Supplementary Figure 2).

Envisioning application of these nanomimics in a biomedical context necessitates vigorous testing of protein fouling, stability in serum, and cytocompatibility.^29^ We utilized fluorescence correlation spectroscopy (FCS) and the related fluorescence cross-correlation spectroscopy (FCCS), which are highly sensitive methods to determine size and concentration (Supplementary Figure 4), loading/release, surface interactions, and enzyme kinetics.^30−33^ First, FCS was performed in 10% (v/v) fetal bovine serum (FBS) with labeled nanoparticles to yield serum stability. Second, randomly labeled FBS components were mixed with unlabeled nanoparticles creating a highly sensitive alternative method for the detection of protein binding. Together, these measurements revealed good colloidal stability for the nanomimics (modified nanoparticles) in the presence of serum and low protein binding over time (Figure 1g and Supplementary Figure 4). In contrast, the unmodified nanoparticles (RCOO^–^) accumulated significant amounts of protein (although much less than cationic nanoparticles), which also caused a significant increase in the observed hydrodynamic diameter (average increase 16 ± 2 nm).

High cytocompatibilities of the nanomimics and the building blocks alone (AMSA and AMBS) were found when tested in two standard cell lines (Figure 1h and Supplementary Figure 5). Only the original carboxylated nanoparticles showed lower compatibility with macrophages, which was independent of the reversible addition–fragmentation chain transfer (RAFT) end groups as demonstrated by removal via aminolysis and thiol-exchange reaction (Supplementary Figures 5 and 6). This confirms the cytocompatible nature of the herein used RAFT-end group and modified nanoparticles in agreement with previous literature.^24,34^ The UV–vis spectroscopic analysis used to follow copolymer modifications, including covalent coupling of aromatic AMBS (Supplementary Figure 6), allowed to estimate that about 30% of the acrylic acid units were modified through our procedure, as expected due to the high density of functional groups. In conclusion, the AMSA- and AMBS-modified copolymers provide serum-stable, low fouling and cytocompatible nanomimics for subsequent applications.

### Polymer–Lipid Coassemblies for Prolonging Blood Residence Time

**Figure 2:**
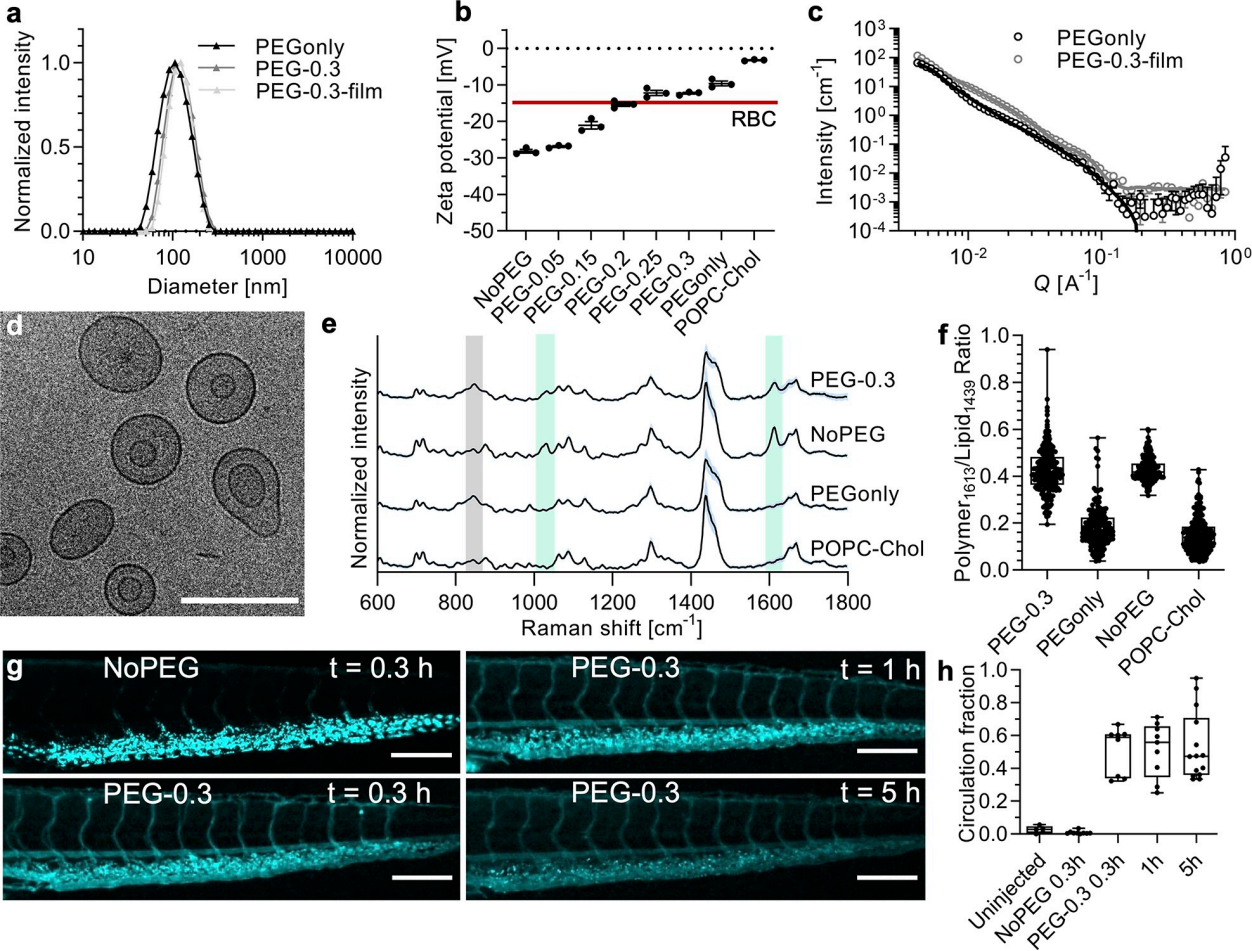
Polymer–lipid nanomimic (PLN) properties. (a) DLS intensity distribution of PEGylated liposomes (PEGonly), and PLNs formed by solvent injection (PEG-0.3) or film rehydration (PEG-0.3-film) technique (average of technical triplicates). (b) Average zeta potential values for various vesicles with increasing amounts of DSPE-PEG5k (mean ± s.d., technical triplicates, red line indicates RBC zeta potential^38^). (c) SANS scattering profiles and fits of PEGonly and PEG-0.3-film vesicles (raw data: open circles; fits: solid lines). (d) Cryo-TEM image of PEG-0.3-film (other images, controls, and statistics are given in Supplementary Figure 8). Scale bar, 200 nm. (e) Normalized Raman intensities of single-particle traps as measured by SPARTA (*n* ≥ 165, mean (black) ± s.d. (light blue)). Shaded gray area highlights PEG (C–O–C skeletal mode at 851 cm^–1^) and shaded light green highlights the copolymer PDLLA-AMBS (1032 and 1613 cm^–1^ corresponding to the sulfonate and amide/aromatic overlapping regions respectively). (f) Raman intensity ratios of the polymer to lipid region (polymer-1613 cm^–1^/lipid-1439 cm^–1^). (g) Fluorescence micrographs of zebrafish embryo tails at time points 0.3 h after NoPEG injection and 0.3, 1, and 5 h after PLN injection (more pictures, videos, and analysis in Supplementary Figure 10 and Supplementary Movies S1, S2, S3, and S4). Scale bars, 200 μm. (h) Quantitative analysis of circulation fraction in zebrafish embryo bloodstream (*n* = 5–14 embryos per group). Box-plots: center line, the median; box limits, upper and lower quartiles; whiskers, minimum and maximum values.

Excess surface charge on nanoparticles is a known factor limiting their ability to function in complex environments and reduce blood circulation times.^35^ To tackle this challenge, we coassembled our AMBS-modified copolymer with lipids, including a PEGylated lipid, to formulate polymer–lipid nanomimics (PLNs) with the aim of partially/transiently passivating the surface and delaying opsonization. Others have previously shown benefits of combining both research spheres to form copolymer/lipid hybrids for other applications.^36,37^ Our PLN design consists of a mixture of PDLLA-AMBS, 1-palmitoyl-2-oleoyl-glycero-3-phosphocholine (POPC), cholesterol, and 1,2-distearoyl-*sn*-glycero-3-phosphoethanolamine-*N*-[methoxy(polyethylene glycol)-5000] (DSPE-PEG5k). PLNs were obtained through film rehydration/extrusion or solvent injection and revealed hydrodynamic diameters of around 100 nm (Figure 2a). The zeta potential could successfully be neutralized by incorporating increasing amounts of PEG-lipid in PLNs (number equals molar fraction of DSPE-PEG5k to vesicle-forming lipid POPC, Figure 2b, Supplementary Figure 7). Controls include PLNs without DSPE-PEG5k (NoPEG), vesicles without the copolymer (mixture of POPC, cholesterol, and DSPE-PEG5k, named PEGonly), and vesicles made from POPC and cholesterol (POPC-Chol).

The zeta potentials of PLNs containing PEG-lipid mole fractions of 0.2–0.3 resemble that of red blood cells (RBCs, - 15 mV),^38^ which was the target value for our herein designed nanomimics. Small-angle neutron scattering (SANS) and cryo-TEM were used to obtain structural information on a bulk and single-particle scale, respectively. The SANS curve for PEGonly (same vesicle composition as PLNs but excluding the copolymer, Figure 2c) has been fitted using a core–shell ellipsoid model. The bulk average shell thickness ranges from 11.8 ± 1.2 Å (equatorial ellipsoid axis) to 5.1 ± 0.6 times this value (polar axis) and reflects the coexistence of spherical liposomes, elongated liposomes, and disc/cylindrical micelle structures. This coexistence of structures agrees well with previous literature SANS studies of DPPC and POPC with PEGylated lipids^39^ and is confirmed by the cryo-TEM data (Supplementary Figure 8).

The PLN SANS data were successfully fitted with a dual layer core–shell model, revealing predominantly liposomal structures with an additional highly hydrated layer. Because of the low contrast between the copolymer and solution scattering, i.e., similar scattering length densities, it is not possible to accurately resolve the copolymer shell on the outer (and potentially inner) lipid membrane without further contrast matching experiments. However, the thickness of this second shell (7.6 ± 0.1 nm) compares well to the hydrophilic polymer size (PAMBS, 4.2 ± 0.1 nm, Figure 1f) when taking into consideration a potentially more stretched conformation on the surface and partial presentation of some polymer toward the PLN core. Cryo-TEM images confirmed the vesicular morphology of PLNs with varying degrees of ellipticity and vesicle-in-vesicle structures (Figure 2d and Supplementary Figure 8). The hydrophobic membrane thicknesses of the nanomimics were similar between SANS (4.1 ± 0.1 nm) and cryo-TEM (5.9 ± 1.0 nm).

To study the coassembly of lipids and copolymers on a single-particle basis, we employed FCCS and the recently developed single-particle automated Raman trapping analysis (SPARTA).^40^ FCCS, which measures the codiffusion of fluorescent species, revealed a high degree of lipid (fluorescent) and copolymer (covalently labeled with fluorophore, here PDLLA-AMSA-CF488) codiffusion when measuring the PLNs compared to control mixtures (Supplementary Figure 7). The label-free SPARTA technique further strengthened the argument for a coassembly since characteristic peaks of lipid, PEG, and copolymer (AMBS modification) appeared in the Raman spectra of single-particle traps in case of PLNs. In contrast, the controls that lacked one or more components did not show any Raman peaks in the corresponding regions. Analyzing the ratio of components across the population of single-particle traps further showed high homogeneity of the samples (Figure 2f).

Both FCS (Supplementary Figure 7) and DLS (Supplementary Figure 7) demonstrated high colloidal stability of PLNs at body temperature and in the presence of serum over time. In contrast, the inverse FCS technique introduced above using unlabeled vesicles and labeled FBS components revealed a gradual increase in protein fouling on the PLNs similar to the PEGonly vesicles (Supplementary Figure 7). We next employed *in vitro* macrophage cultures to study the effect of PLN PEGylation on cell association in the presence of serum proteins, where we expected a delayed interaction for formulations incorporating more PEG-lipid.^41^ We did not observe any cytotoxic effect for PLNs, and there was a clear inverse dependence of PEG-lipid amount versus cellular association (Supplementary Figure 9). This confirms the benefit of incorporating the passivating PEG-lipid in the PLN formulation. Since sulfonated compounds/nanoparticles mimic the structure of heparin, they could potentially function as an anticoagulant. Hence, we also conducted antifactor Xa activity tests. These revealed only negligible anticoagulation activity for the samples (0.02%, 0.23%, and 0.48% for AMSA-, AMBS-nanomimics, and PLNs, respectively, compared to heparin on a weight basis, Supplementary Figure 9), allowing a high dose to be administered without reaching the levels needed for an anticoagulation effect.

Finally, to explore the properties of these nanomimics *in vivo*, we used zebrafish embryos to probe the blood circulation characteristics of the nanomimics. The zebrafish model has recently been deemed as a valuable *in vivo* model which correlates well with rodent data, with the advantage of tissue transparency for ease of in-line microscopy imaging at low cost.^42−45^ Incorporating PEG-lipid in the PLN formulations clearly extended the blood circulation time, compared to PLNs without PEG (Figure 2g,h) and polymer nanomimics (Supplementary Figure 10).

There is evidence that protein adsorption is necessary for the stealth effect of PEGylated nanoparticles.^41^ This could explain the observations made herein with respect to time-dependent protein fouling on PLNs in the FCS experiments (Supplementary Figure 7) corresponding to long blood residence times (Figure 2g,h). Another possibility is a transient blocking mechanism, with PLN components exchanging with serum components over time, as for example found for PEGylated lipid nanoparticle formulations of RNA.^46,47^ By extending the circulation time of surface-active nanoparticles, our strategy of copolymer–lipid coassembly provides a major step forward in the development of nanoparticles with broad-spectrum utility against pathogens *in vivo*.

### Polymer Nanomimics Are Potent Virustatic Entry Inhibitors of HSV-2 and SARS-CoV-2

**Figure 3:**
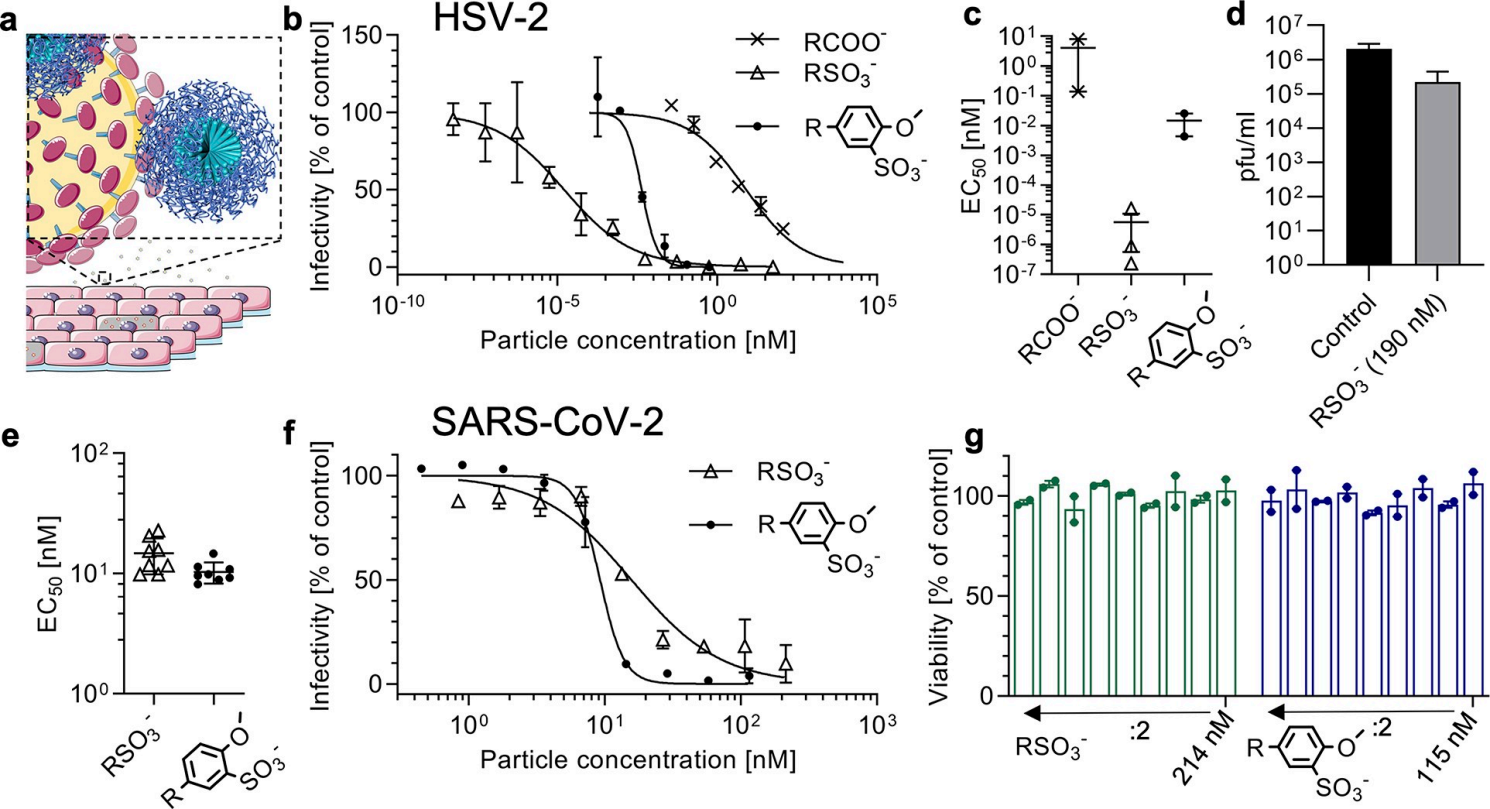
Virus inhibition by polymer nanomimics. (a) Schematic of virus inhibition by polymer nanomimics. Schematic modified from the Servier Medical Art Web site CC-BY. Nanoparticle schematic reproduced from ref (26) with permission. Copyright 2016 the Royal Society of Chemistry. (b) Dose–response curves for HSV-2 inhibition using various polymer nanomimics: PAA EC_50_ 8.0 nM (4.9 μg/mL); AMBS EC_50_ 4.4 pM (11 ng/mL); AMSA EC_50_ 16 fM (21 pg/mL) (mean and range of *N* = 1 independent experiment with technical duplicates). (c) Obtained EC_50_ values from dose–response curves as shown in (b) (mean ± s.e.m., *N* ≥ 2 independent experiments with technical duplicates). (d) Virucidal test with HSV-2 and AMSA nanomimics. (e) EC_50_ values (mean ± s.d., *N* = 8 independent experiments with technical duplicates) obtained from dose–response curves, testing polymer nanomimics against SARS-CoV-2 infection (variant B.1) in Vero cells. (f) Example dose–response curves from (e): AMBS EC_50_ 9 nM (22 μg/mL); AMSA EC_50_ 16 nM (20 μg/mL) (mean and range of *N* = 1 independent experiment with technical duplicates). (g) Cytocompatibility of nanomimics with Vero cells tested at same concentrations and incubation times as in f (*N* = 1 independent experiment with technical duplicates).

Various compounds, polymers, and nanoparticles that present or mimic host cell receptors, such as heparan sulfate and sialic acid, are widely known to have antiviral properties.^48,49^ We next sought to test whether our polymeric version of short chain sulfonates on the surface of our polymeric nanoparticles (Figure 1a) might produce a potent virus inhibitory nanoparticle. When tested on HSV-2 with epithelial host cells, polymeric nanomimics were found to be extremely potent virus entry inhibitors (Figure 3a–c). The best nanomimics (AMSA) revealed an average half maximal effective concentration (EC_50_) of 5.8 fM (9.5 pg/mL of anionic polymer, Figure 3c). This is many orders of magnitude more potent than other inhibitors such as heparin (EC_50_ 56 nM, 1 μg/mL) and multivalent gold nanoparticles (AuNPs: EC_50_ 5.3 nM, 1.6 μg/mL),^4^ dendrimers (EC_50_ 130 nM, 1.3 μg/mL),^5^ or nanogels (EC_50_ 90 μg/mL),^6^ both, when compared on the molar and weight scale, respectively. The potency of these nanomimics can be explained by the flexible polymer chains, with repetitive sulfonates presented along the side chains, extending from the PLA core. This likely allows conformational flexibility and optimal multivalent binding when interacting with the viral envelope proteins, in contrast to more rigid presentations of single sulfonate groups or short dendritic sulfonates on other types of nanoparticles. Exposing a methoxy-benzene based sulfonate (AMBS) on our nanomimics created less potent inhibitors versus a simpler sulfonate (AMSA), in agreement with the literature,^50^ which could be leveraged to tune the nanomimics toward specific pathogens.

Similar to the design of virucidal AuNPs,^4^ we formulated another version of our nanomimics with 11-mercaptoundecanesulfonate (MUS) ligands exposed at the end of the copolymers through aminolysis and a thiol-exchange reaction (Supplementary Figures 6 and 11). As expected, AMSA- and AMBS-based nanomimics did not show any virucidal activity (Figure 3d and Supplementary Figure 11), but also the MUS-modified version failed to produce a virucidal effect (Supplementary Figure 11). This absence of a virucidal mechanism of action is most likely attributed to a relatively low density of MUS on our nanomimics (estimated to about 50 per particle) and a more flexible polymer layer, which might not be able to exert sufficient force to deform the virus particles as described for the MUS-AuNPs.^4^

To test the broad utility of our platform, we next tested AMSA- and AMBS-modified nanomimics against SARS-CoV-2 entry into epithelial cells. As with HSV-2, these nanomimics were found to be inhibitory (Figure 3e,f), although only at higher doses compared to HSV-2 (Figure 3b,c). However, the level of activity of our nanomimics seen against SARS-CoV-2 is still an improvement in inhibitory activity, when compared to inhibition with heparin in our assays (Supplementary Figure 11, EC_50_ ≈ 500 μM or 8.9 mg/mL, unfractionated heparin, UFH, ∼ 18 kDa); EC_50_ ∼450 times (AMSA, EC_50_ 15 nM or 19 μg/mL of anionic polymer) and ∼350 times lower (AMBS EC_50_ 10 nM or 24 μg/mL) based on a weight scale. This again demonstrates the benefit of using a multivalent nanoparticle versus a simple polymer (heparin). Previous literature has shown heparin-based SARS-CoV-2 inhibition *in vitro*,^7,51−54^ but a large variation in potency has been reported (EC_50_ from 6 ng/mL to >1000 μg/mL),^8,55^ while it needs to be highlighted that inhibition is highly dependent on the molecular weight of heparin and whether live virus or pseudovirus is used. Pseudovirus was inhibited at low concentrations of UFH, with EC_50_ at 6 ng/mL.^51^ Low molecular weight heparin (LMWH) was much less potent than UFH against live virus (EC_50_ at 3.4–7.8 mg/mL).^52^ For UFH against live virus, several publications have shown EC_50_ values in the low tens of μg/mL,^7,52,54^ but one other study failed to achieve any inhibition when tested up to 1000 μg/mL,^8^ which is in agreement with our results (EC_50_ at 8.9 mg/mL). Variations in SARS-CoV-2 isolates used, their eventual cell adaptation, and differences in host cell lines could potentially be causes for this high variability.^55^ Other causes could be the type of inhibition assay, dose of virus used, and the source of heparin, but further investigations that are beyond the scope of this study are necessary. We have also cross-validated our assay with a standard from the WHO Reference Panel (anti-SARS-CoV-2 immunoglobulins, NIBSC code: 20/150),^56^ which showed inhibitory activity, and hence confirms the high robustness of our assay (Supplementary Figure 11).

Although mainly designed for the antimalarial application, we tested our hybrid PLNs against SARS-CoV-2, and they were found to be active but less potent than our polymer nanomimics (Supplementary Figure 11). This could be explained by the lower affinity of PLNs to viruses due to the incorporated PEG, which might disturb the interaction between the virus and PLN surface. Using a less-specific nanoparticle-based approach to inhibit viruses, e.g., when compared to target specific antibodies, is accompanied by a theoretical lower sensitivity to virus mutations.^48^ The Beta variant B.1.351 already shows reduced susceptibility to neutralization by vaccine sera and convalescent plasma.^57^ In contrast, we found similar activity of our nanomimics also against this variant (Supplementary Figure 11), which might suggest a higher robustness of these less specific inhibitors with respect to mutating viruses. The necessity of further optimizations, ideally turning our nanomimics virucidal, and the lengthy regulatory approval process will not allow timely development of such a treatment for the current pandemic.^58^ However, these data provide a basis for the development of simple, broad-spectrum synthetic antivirals, as alternatives of cell-membrane based nanodecoys,^11^ which is highly desirable to prepare for future pandemics.^59^

### Nanomimics Inhibit Malaria Parasites *In Vitro* and *In Vivo*

Given the analogous mechanisms of initial host cell interaction by viruses and malaria parasite (*Plasmodium*) blood-stage merozoites, but the known loss of invasive capacity seen with extracellular parasites after they leave their host cell,^60,61^ we sought to test our nanomimics for their application in a non-biocidal capacity. Parasite membrane disruption by an inhibitory nanoparticle would not be necessary, since blocking or disturbing the invasion process for a few minutes will be sufficient to yield merozoites that are devoid of any invasive potential. Hence, we explored the application of our nanomimics, which expose synthetic mimics of RBC invasion receptors on the surface, against malaria parasites. We first tested the potency of our nanomimics for inhibiting parasite growth *in vitro* using various parasite strains of *Plasmodium falciparum*, the species responsible for most mortality to malaria.^1^ In addition, we also tested our nanomimics against zoonotic *P. knowlesi* parasites, recently transferred to culture in human RBCs,^62^ and representing a growing problem. Parasitemia (% of infected RBCs) was measured by flow cytometry (Supplementary Figure 12) after the parasites went through one cycle of host cell egress and reinvasion of healthy RBCs to form ring stage parasites in the presence and absence of inhibitors.

**Figure 4:**
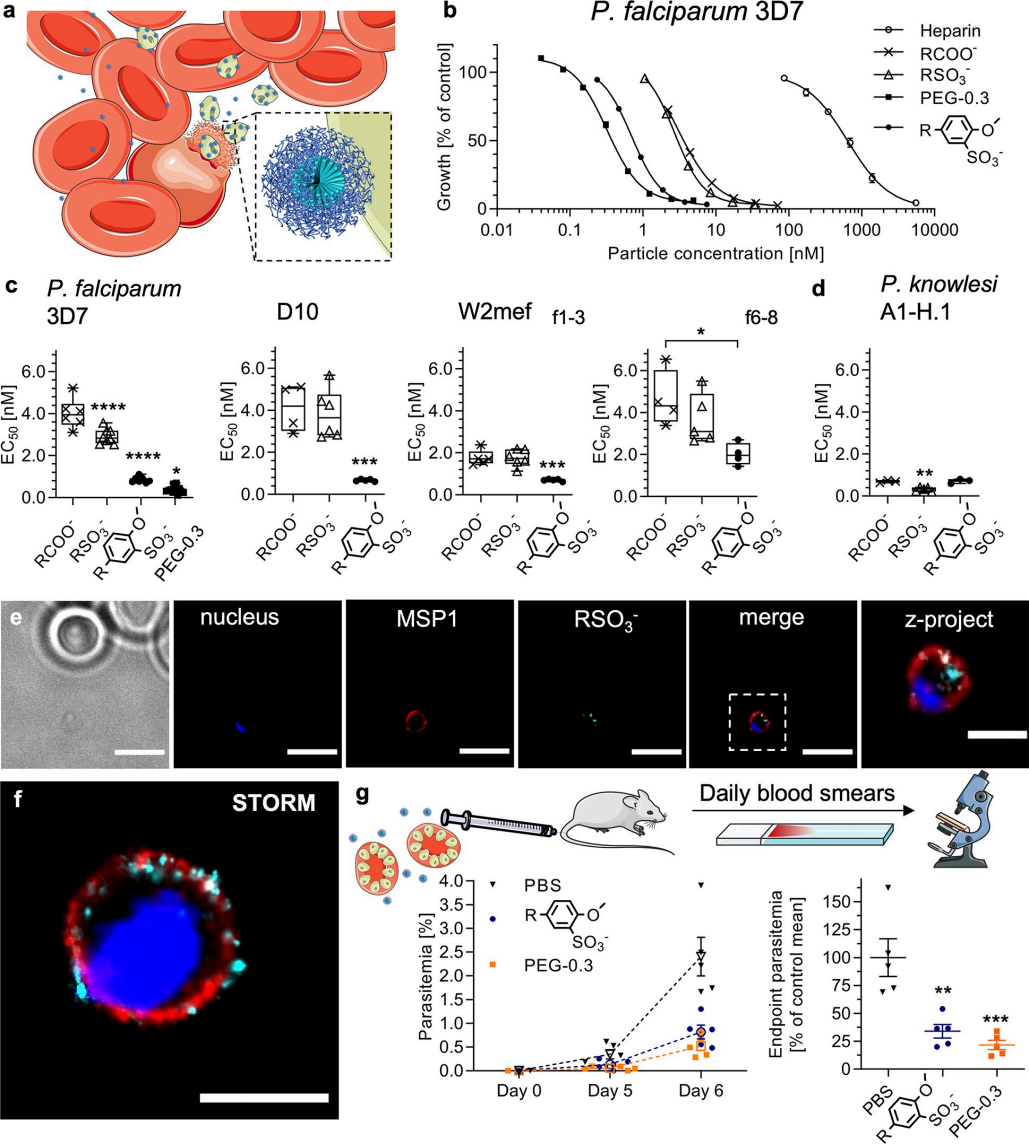
In vitro and in vivo malaria parasite inhibition with nanomimics. (a) Schematic of *Plasmodium* merozoite (green) inhibition of RBC (red) invasion by nanomimics (blue). Schematics modified from the Servier Medical Art Web site CC-BY. Nanoparticle schematic reproduced from ref (26) with permission. Copyright 2016 the Royal Society of Chemistry. (b) Dose–response curves for *P. falciparum* 3D7 inhibition in suspension culture: Heparin EC_50_ 640 nM (12 μg/mL); PAA EC_50_ 3.6 nM (2.2 μg/mL); AMSA EC_50_ 2.7 nM (3.5 μg/mL); AMBS EC_50_ 0.7 nM (1.7 μg/mL); PEG-0.3 EC_50_ 0.3 nM (0.7 μg/mL) (mean and range of *N* = 1 independent experiment with technical duplicates). (c, d) EC_50_ values obtained from dose–response curves using various *P. falciparum* strains (3D7, D10, W2mef) and *P. knowlesi* A1-H.1 strain (*N* ≥ 3 independent experiments with technical duplicates, one-way ANOVA with Tukey’s multiple comparisons test, *P < 0.05, **P < 0.01, ***P < 0.001, ****P < 0.0001). (e) Widefield deconvolution imaging (middle slice of z-stack) of nanomimic-inhibited merozoites (nucleus in blue, MSP1 in red, PDLLA-AMSA-CF488 in cyan) and zoomed z-projection of z-stack. Scale bars, 5 and 2 μm (zoom), respectively. (f) STORM image of nanomimic- (PDLLA-AMSA-Cy5, cyan) inhibited merozoite (nucleus in blue, MSP1 in red; separate images in Supplementary Figure 14). Scale bar, 1 μm. (g) Schematic of *P. berghei in vivo* experiment. Conditions were 1 × 10^5^*P. berghei*-infected RBCs (at schizont stage) and 1.5 mg/kg treatment on day 0. Parasitemia followed over time (dotted lines and open symbols represent mean ± s.d., *n* = 5 mice per group) and corresponding plot of % inhibited vs PBS control at day 6 when end point parasitemia >1% for PBS group (one-way ANOVA with Tukey’s multiple comparisons test, **P < 0.01, ***P < 0.001, comparison to PBS control shown). Box-plots: Center line, the median; box limits, upper and lower quartiles; whiskers, minimum and maximum values. Schematics modified from the Servier Medical Art Web site CC-BY.

All the polymer nanomimics were potent inhibitors of malaria parasite invasion of RBCs, with the highest performing exhibiting an EC_50_ of 0.67 ± 0.05 nM (1.6 ± 0.1 μg/mL, D10, Figure 4a–d). Some differences were observed depending on the type of anionic compound presented on the surface and invasion pathways used by the different strains. The parasite strain W2mef, which uses a sialic-acid-dependent invasion pathway, can be inhibited with lower concentrations of nanomimics compared to sialic acid-independent strains (3D7 and D10). This can be explained by W2mef’s dependence on more charged residues (heparan sulfate and sialic acid) for successful invasion making a charge-based inhibition more potent. In contrast to the viral inhibition data of HSV-2 that showed lower potency of the AMBS-based nanomimics, all *P. falciparum* strains were inhibited most efficiently with these nanomimics. Activity with high potency was also confirmed against zoonotic *P. knowlesi* cultured in human RBCs (Figure 4d).

Smaller particles (f6–8) were less potent than the main ones used herein (f1–3), while the largest of all, PLNs (PEG-0.3), provided the best activity (EC_50_ at 0.38 ± 0.16 nM, 0.9 ± 0.4 μg/mL). This inhibitory potency of PLNs is a substantial improvement compared to heparin-based inhibition (EC_50_ at 640 nM, 12 μg/mL), while additionally providing the urgently required properties such as negligible anticoagulation activity, long blood circulation time, and multifunctionality (potential to load hydrophilic and hydrophobic compounds in the future, for example, for downstream immunomodulation). The high potency of PLNs contrasts the virus data (Figure 3), which showed reduced activity of PLNs. Again, the poor extracellular survival of merozoites, the type of assay, and a dynamic PLN structure are possible explanations for this phenomenon. By including cationic moieties into the polymer nanoparticles, the antiplasmodial activity was greatly reduced, while rod-shaped nanoparticles were less potent than the spherical ones (Supplementary Figure 12). Additionally, nanomimic potency decreased upon an increase in spacer length of the amino-sulfonate molecules (Supplementary Figure 12). The virucidal MUS-AuNPs^4^ tested against parasites showed much lower potency compared to polymer nanomimics, highlighting differences in virus versus parasite inhibition (Supplementary Figure 12). The building block AMSA alone had some activity at high concentrations, while AMBS was inactive (Supplementary Figure 12). To avoid any influence of excess reagents, they were always removed from the nanoparticles by sequential SEC (Supplementary Figures 3 and 6).

Live imaging of the inhibitory process *in vitro* revealed surface-binding and blockage of merozoites by nanomimics after and even during egress (Supplementary Figure 13 and Movie S5). This suggests invasion inhibition as the mode of action, as found previously for other similar heparin-based structures.^21^ Partial access of the nanomimics to the intracellular space of the host cell just before complete merozoite egress could explain the high potency of our nanomimics that have to compete with uninfected RBCs presented to merozoites after egress. More detailed imaging of nanomimic-inhibited merozoites by widefield deconvolution and stochastic optical reconstruction microscopy (STORM) revealed nanomimic binding to the major surface protein 1 (MSP1) layer that coats the whole merozoite surface (Figure 4e–f, Supplementary Figure 14). MSP1 and many other merozoite invasion ligands are known to interact with heparin/heparan sulfate,^63^ providing a multitude of targets for our nanomimics. When comparing our nanomimics to more specific inhibitors, e.g., antibodies against merozoite surface ligands, our inhibitory capacity is high. Many antibody studies have shown very low potency, with one of the best ones against *P. falciparum* reticulocyte binding protein homologue 5 (*Pf*Rh5)—the current frontrunner in blood-stage vaccine development—requiring about 400 nM for EC_50_.^64^ In comparison, our nanomimics are only approximately four times larger than an antibody but have a much higher inhibitory potency (>2 orders of magnitude lower EC_50_ values) as well as a simple architecture.

We next performed initial studies to see whether these nanomimics are applicable *in vivo.* After intravenous (i.v.) administration of nanomimics alone, histopathology analysis did not reveal any obvious alterations in any of the major organs when compared to the PBS control (Supplementary Figure 15). To test the *in vivo* efficacy of nanomimics, the rodent malaria mouse model *P. berghei* ANKA was used with synchronized late-stage parasites (schizonts) coinjected together with the nanomimics to synchronize the infection, as it is the case in a human infection, and to reduce the influence of the circulatory behavior of our nanomimics in this first proof-of-concept (Figure 4g). In this scenario, all the parasites will reinvade new host RBCs within 2–4 h after injection.^65^ Both AMBS-modified nanomimics and PLNs (PEG-0.3) successfully inhibited parasites in the rodent model (Figure 4g), while AMSA-modified nanomimics at the same dose were inactive (Supplementary Figure 16), mirroring the *in vitro* data (Figure 4b,c). When compared to the PBS control, the end point parasitemia (when parasitemia >1% for PBS group) at day 6 was significantly reduced (about 75%). This means approximately three-quarters of the parasite inoculum was inhibited by the functioning nanomimics on day 0. Given the difficulty to get any *in vivo* inhibition with vaccine-induced or passively transferred antibodies against *P. berghei* merozoite proteins,^66^ our data of partial inhibition with nanomimics suggest a benefit of using less-specific multivalent inhibitors.

This proof-of-concept study sets the basis for thorough future investigations of administration timing and accompanying downstream effects on the immune system. Incorporation of immunomodulatory molecules in the nanomimics, subsequently bound to whole parasites, is a further means of tuning the immune response. The time scales of PLN activities will have to be tested in detail to establish the exact time windows for parasite inhibition. However, this optimization should ideally be performed with advanced mouse models using human parasites *P. falciparum*,^67^ due to the differences in the parasite life cycle and extracellular merozoite survival between *P. falciparum* and *P. berghei*.

Since we envision application of this strategy for immunomodulation to boost the response against extracellular parasites, a complete inhibition is not necessary and in fact not desirable since we want to avoid overwhelming the immune system. Our reduction of parasite numbers by about 75% is sufficient for these future studies. Even these percentages of inhibition provide huge numbers of arrested merozoites (here ca. 10^6^ merozoites inhibited, when assuming 12–16 merozoites per schizont^65^) for the proposed downstream effects; the remaining parasites can simply be eliminated with the addition of conventional antimalarials that kill intracellular parasites. These future works will establish the applicability of nanomimic-inhibited parasites for immunomodulation to better protect from subsequent infections, which could be a major new technology to be added to the antimalarial arsenal.

## Conclusions

We have presented here a comprehensive framework for the design of highly potent pathogen entry inhibitory nanomimics, with activity in HSV-2 and SARS-CoV-2 virus as well as malaria parasite infection models. The highly potent HSV-2 inhibition encourages further efforts to develop virucidal formulations of nanomimics at ultralow, fM concentrations, which might provide novel treatment options in future emergent pandemics. Potent malaria parasite inhibition with our nanomimics *in vitro* and *in vivo* paves the way for potential future use of a nanomedical approach against malaria. Demonstration of prolonged blood circulation time by coassembly of copolymers, presenting the inhibitory sulfonates, together with lipids and PEG-lipid, is a major step forward for use of these types of inhibitors systemically since polyanions are typically known to have short circulation half-lives.^20^ Further variation of lipid components, copolymer types, and carbon chain lengths of the PEG-lipids^46^ is a future means of optimizing the partial and/or transient PEG blocking on the PLN surface. Extending this technology, it is conceivable that the nanocarrier compartment could additionally be loaded with drug molecules and/or immunomodulators for controlled delivery in the future. In addition, *in situ* inhibition of whole pathogens and subsequent delivery of these nanomimic-pathogen complexes to sites of immune recognition might provide an alternative strategy to increase protection from future infections. Although thorough *in vivo* investigations, including further evaluation of potential side effects due to the nonspecific nature of our inhibitors, are needed to establish applicability of these concepts, they could provide urgently needed alternative tools to tackle the huge challenge of current and emerging infectious diseases.

## Materials and Methods

All of the data were plotted using GraphPad Prism 9.0.0.

### Polymer Nanoparticle Formation and Modification

Polymer nanoparticles were formed by either bulk hydration or solvent injection method. In bulk hydration, the block copolymer PDLLA-*b*-PAA (poly(dl-lactide-*block*-acrylic acid), 9 kDa-9 kDa, Sigma-Aldrich, 802190) was dispersed at 10 mg/mL in MES buffer (0.5 M MES (Sigma-Aldrich) and 0.25 M NaCl (VWR), pH 6.0) by vigorous stirring for 30 min and additional ultrasonication (bath) for 45 min. The same procedure was used for the related copolymer PLLA-*b*-PAA (poly(l-lactide-*block*-acrylic acid), 4.5 kDa-18 kDa, Sigma-Aldrich, 805718) and homopolymer PAA (poly(acrylic acid), 10 kDa, Sigma-Aldrich, 775843). The latter yielded controls of the hydrophilic block alone at a comparable length compared to PDLLA-*b*-PAA. In the solvent injection method, the polymer PDLLA-*b*-PAA (9 kDa-9 kDa, Sigma-Aldrich) was dissolved in THF at 100 mg/mL and injected rapidly into stirred MES buffer to yield a final solution of 10 mg/mL after evaporating THF by means of open-cap stirring and blowing a N_2_-stream above the surface for about 30 min.

Polymer nanoparticle modification was performed by mixing the desired amino-molecule with the 10 mg/mL nanoparticle solution in MES buffer. For 10 mg of block copolymer, the following equivalents with respect to the number of acrylic acid (AA) units were used for homo modifications: 2.5 equiv of AMSA (aminomethanesulfonic acid, 19.2 mg, Sigma-Aldrich, 127442); 1.5 equiv of AMBS (5-amino-2-methoxybenzenesulfonic acid, 21.4 mg, ChemCruz, SC-233225). For heteromodifications with combinations of amino-molecules, the ratios stated in the figures were used (equiv given corresponds to the cationic molecule): T (taurine, Sigma-Aldrich, T8691); AES (2-aminoethyl hydrogen sulfate, Sigma-Aldrich, 06720); HT (homotaurine, Sigma-Aldrich, A76109), ATA ((2-aminoethyl)trimethylammonium chloride hydrochloride, Sigma-Aldrich, 284556); DMAPA (3-(dimethylamino)-1-propylamine, Sigma-Aldrich, D145009); DMEDA (*N,N*-dimethylethylenediamine, Sigma-Aldrich, D158003); EDA (ethylenediamine, Sigma-Aldrich, E26266). After the molecules were dissolved in the nanoparticle solution, an aliquot of 0.5 equiv of EDC-HCl in 12 μL of MES buffer (*N*-(3-(dimethylamino)propyl)-*N′*-ethylcarbodiimide hydrochloride, 6.6 mg, Sigma-Aldrich, E7750) was added, and the solution was stirred vigorously at r.t. The EDC-HCl stock was prepared freshly from powder for each time point. After about 0.5–1 h, the sample was ultrasonicated (bath) for 1 min before adding another 0.5 equiv EDC-HCl aliquot; in total, eight additions of 0.5 equiv of EDC-HCl over the period of about 6–8 h were made. Post modification, the sample was purified and separated based on size using size exclusion chromatography (SEC). First, the sample was run through a PD MidiTrap column (GE Healthcare) equilibrated in phosphate buffer (0.1 M phosphate (Sigma-Aldrich), 0.05 M NaCl (VWR), pH 7.4). Second, the sample was run through a 30 cm Sepharose 6B column (Sigma-Aldrich, 6B100) equilibrated in phosphate buffered saline (PBS, Sigma-Aldrich, D8537). Desired fractions were pooled and sterile filtered by passing through a 0.22 μm syringe filter (Millipore, SLGV013SL) inside a biosafety cabinet and stored at 4 °C.

Covalent modification with fluorescent dyes: prior to AMSA modification (see exact protocol above), Sulfo-Cyanine5 amine (Lumiprobe, 233C0) or CF 488A amine (Sigma, SCJ4600014-1MG) was conjugated first. The desired fluorescent dye was dissolved in DMSO at 10 mM and 41.6 μL added to 1 mL of 10 mg/mL nanoparticle solution in MES buffer (see above). Four additions of 0.25 equiv of EDC-HCl in 6 μL of MES buffer (3.3 mg, Sigma-Aldrich, E7750) over the course of about 2 h was followed by addition of AMSA (see above), and the modification reaction was continued and purified as described above.

For further use of the modified copolymers for integration in lipid vesicles (see below), the modified polymer nanoparticles after passing through the PD MidiTrap column (see above) were passed through a PD10 (GE Healthcare) column equilibrated in ddH_2_O. Typically, 5 mL of nanoparticle solution was passed through 5 PD MidiTraps and pooled to subsequently pass through 3 PD10s, yielding 10.5 mL final volume. The sample was concentrated to about 3 mL using Amicon 100 kDa ultracentrifugation device (Sigma). A column was packed with about 3–4 mL of ddH_2_O washed cation exchange resin (AG-50W-X8, H^+^ form, Bio-Rad, 1435451), and the concentrated sample was run through to exchange the cationic counterions with protons. The sample (PDLLA-AMBS or PDLLA-AMSA) was sterile filtered (0.22 μm syringe filter) and freeze-dried thereafter to yield a white fluffy powder.

To yield sterile samples for *in vivo* testing, MES buffer and phosphate buffer were sterile filtered (0.22 μm syringe filter) and were subsequently passed through an Amicon 3 kDa ultracentrifugation device and the washthrough used. Sterile plastic vials were used for bulk hydration nanoparticle formation and subsequent modification (see procedure above). Stirring bars were cleaned by soaking in 0.5 M NaOH overnight and washed with sterile PBS (Sigma-Aldrich, D8537). All of the columns were washed in 0.5 M NaOH for 4 h and equilibrated with sterile buffers before purifying the nanoparticle samples (columns were run in a biosafety cabinet), which were finally sterile filtered again (0.22 μm syringe filter) before injection.

### Aminolysis and End Group Modification

A total of 150 mg of PDLLA-*b*-PAA (9 kDa-9 kDa, Sigma-Aldrich, 802190) and 72 mg of DTP (2,2′-dithiodipyridine, Sigma-Aldrich, D5767) were dissolved in 7 mL of DMF and transferred to a 25 mL glass round-bottom flask and sealed with a septum. After 15 min of degassing by N_2_ bubbling, a large excess of butylamine (BA, Sigma-Aldrich, 471305) in DMF (1.7 mL of a 10% (v/v) BA solution in DMF, 15 min N_2_ degassed) was added under stirring. The reaction was stirred under continuous N_2_ bubbling for 3 h, with brief heating with a heat gun once the solution became too viscous. The crude reaction mixture was precipitated in ice cold diethyl ether three times, with 2 min centrifugation 2000 RCF to collect the precipitate, which was subsequently dried under vacuum. The precipitate was subsequently hydrated directly in MES buffer (1 M MES, 0.5 M NaCl, pH 6.0) to form nanoparticles at 38 mg/mL through 30 min vigorous stirring. A total of 0.6 mL of this solution was subsequently transferred to an excess amount of SMES (9 mg, Sodium 2-mercaptoethanesulfonate, Sigma-Aldrich, M1511) or MUS (0.7 mg, 11-mercapto-1-undecanesulfonate from ref (4)) and stirred at r.t. for 2.5 h. UV–vis spectroscopy (SpectraMax M5, Molecular Devices) was used to follow the exchange reaction. A control without addition of a thiol molecule and controls of nanoparticle solutions after modification (see above) were included to show successful removal of the RAFT end group, DTP modification, and finally exchange with SMES/MUS. The end group-modified nanoparticle solution was then sequentially passed through a PD MiniTrap an PD MidiTrap column (GE Healthcare) equilibrated in MES buffer (0.5 M MES, 0.25 M NaCl, pH 6.0). Subsequent modification of the acrylic acid units was performed as described above.

### Polymer Amount Quantification

Farndale microassays^21,28,68^ were performed to quantify the amount of functional (sulfonated) polymer in the final, purified nanoparticle solutions. The DMMB (1,9-dimethyl-methylene blue zinc chloride double salt, Sigma-Aldrich, 341088) solution was prepared as suggested elsewhere.^68^ A total of 250 μL of this DMMB solution was added into 96-well plates followed by the addition of 50 μL of a concentration series of corresponding reference polymers in PBS. PSS (1 MDa, poly(sodium 4-styrenesulfonate) solution, Sigma-Aldrich, 527491) was used for AMBS-modified nanoparticles, while PAMPS (2 MDa, poly(2-acrylamido-2-methyl-1-propanesulfonic acid) solution, Sigma-Aldrich, 191973) was used as reference for AMSA-modified nanoparticles. Absorbance at 525 nm (PAMPS) or 590 nm (PSS) was measured immediately after mixing the samples (SpectraMax M5, Molecular Devices). Diluted nanoparticles samples in PBS were added for subsequent quantification through subtraction of the PBS control, linear regression of the calibration data, and interpolation of the unknown nanoparticle solutions. These concentrations of functional/active polymer were subsequently used as a reference in all of the assays.

A similar toluidine blue (TB) microassay was performed to quantify the amount of poly(acrylic acid) in unmodified nanoparticle solutions. A 100 μM solution of TB (Sigma-Aldrich, 89640) in ddH_2_O was prepared. A total of 270 μL of this TB solution was pipetted into 96-well plates followed by addition of 30 μL of poly(acrylic acid) standards (250 kDa, Sigma-Aldrich, 416002) or purified nanoparticle solution in PBS. Absorbance (625 nm) was measured immediately after mixing the samples (SpectraMax M5, Molecular Devices). Diluted nanoparticles samples in PBS were added for subsequent quantification through subtraction of the PBS control, linear regression of the calibration data, and interpolation of the unknown nanoparticle solutions.

### Polymer–Lipid Nanomimic (PLN) Assembly

PLNs were assembled through film rehydration or the solvent injection method. For film rehydration, lipids in chloroform (25 mg/mL) were mixed, and PDLLA-AMBS (freeze-dried after ion-exchange, see above) in ethanol was added. As an example, 2.7 mg of 1-palmitoyl-2-oleoyl-glycero-3-phosphocholine (POPC, Avanti, 850457P-200 mg), 1.4 mg of cholesterol (Sigma-Aldrich, C8667–5G), 8.6 mg of 1,2-distearoyl-*sn*-glycero-3-phosphoethanolamine-*N*-[methoxy(polyethylene glycol)-5000] (DSPE-PEG5k, Laysan Bio, MPEG-DSPE-5000-1g), and 11.4 mg of PDLLA-AMBS were combined (PEG-0.3). The number at the end of the PLN names corresponds to the molar fraction of DSPE-PEG5k to vesicle-forming lipid POPC. Samples without DSPE-PEG5k or without PDLLA-AMBS were termed NoPEG or PEGonly, respectively. To obtain fluorescent PLNs, 45 μg of fluorescent lipid 1,1′-dioctadecyl-3,3,3′,3′-tetramethylindodicarbocyanine perchlorate (DiD, Thermo Fisher Scientific, D307) or 3,3′-dioctadecyloxacarbocyanine perchlorate (DiO, Thermo Fisher Scientific, D275) was added if necessary. For FCCS studies, PDLLA-AMSA-CF488 (freeze-dried after ion-exchange, see above) was used instead of PDLLA-AMBS. The solvent was evaporated by hand using a N_2_-stream, while the glass vial was rotated. The film was further dried by desiccation in a vacuum chamber overnight. The film was hydrated in phosphate buffer (0.1 M phosphate, 0.05 M NaCl, pH 7.4) and pH adjusted to 7.4 with drops of 1 M NaOH if necessary. The solution was stirred vigorously for about 2–3 h, followed by extrusion through 0.4 μm (4×), 0.2 μm (10×), and 0.1 μm (21×) polycarbonate membranes using a mini-extruder (Avanti, Sigma-Aldrich, 610000). The extruded solution was subsequently run through a 30 cm column filled with Sepharose 2B-CL (Sigma-Aldrich, CL2B300) equilibrated in PBS. For the solvent injection method, the same amounts of lipids (see above) were dissolved in ethanol and mixed with PDLLA-AMBS in ethanol (total, final volume of 0.4 mL). This ethanolic mix was injected in one go into 0.6 mL vigorously stirred phosphate buffer (0.1 M phosphate, 0.05 M NaCl) and pH adjusted with drops of 1 M NaOH if necessary. Ethanol was subsequently evaporated with a N_2_ stream (about 2 h) before running the sample through a 30 cm column filled with Sepharose 2B-CL (Sigma-Aldrich, CL2B300) equilibrated in PBS.

To yield sterile samples for *in vivo* testing, phosphate buffer (0.1 M phosphate, 0.05 M NaCl) was sterile filtered (0.22 μm syringe filter) and was subsequently passed through an Amicon 3 kDa ultra centrifugation device and the washthrough used. Stirring bars were cleaned by soaking in 0.5 M NaOH overnight and washed with sterile PBS (Sigma-Aldrich, D8537). All the columns were washed in 0.5 M NaOH for 4 h and equilibrated with sterile PBS (Sigma-Aldrich, D8537) before purification of the nanoparticle samples (columns were run in a biosafety cabinet). Samples were concentrated using an Amicon 100 kDa ultra centrifugation device and were finally sterile filtered (0.22 μm syringe filter) before injection.

### DLS and Zeta Potential Measurements

Measurements (*n* = 3) were performed on a Malvern Zetasizer Nano-ZS. A total of 70 μL of nanoparticle suspension in PBS was typically used in single-use microcuvettes. For zeta potential measurements, 950 μL of ddH_2_O (for nanoparticles) or 950 μL of 0.3 M sucrose (for PLNs) was mixed with 50 μL of purified sample solution in PBS.

### TEM and cryo-TEM

TEM grids (Electron Microscopy Sciences, CF200-Cu, 215-412-8400) were plasma cleaned for 1 min before adding 5 μL of nanoparticle solution in PBS. The nanoparticle solution was kept on the grid for 1 min before blotting away the liquid. The samples were washed with two drops of ddH_2_O before two drops of negative stain were applied (2 wt % uranyl acetate in water, 0.45 μm filtered), the second drop kept on the sample for 15 s before being blotted away. The grids were left to dry overnight before imaging on a JEOL 2100F.

For cryo-TEM, 3 μL of sample in PBS was applied on a glow-discharged Quantifoil R2/2 grid (400 copper mesh, Quantifoil Micro Tools GmbH, Großlöbichau, Germany), which contains a thin continuous carbon layer on top. Samples were prepared with an automatic plunge freezer FEI Vitrobot (Thermo Fisher Scientific, Waltham, MA, USA) operated at 100% relative humidity at 21 °C. In brief, the sample was incubated on the grid for 10 s before blotting for 4 s and plunging it into liquid ethane. These specimens were then imaged on a JEOL JEM-2100f transmission electron microscope (JEOL Ltd., Tokyo, Japan) using a TVIPS TemCam-XF416 CMOS camera (Tietz Video and Image Processing Systems GmbH, Gauting, Germany).

### FCS and FCCS

Nanoparticles/PLNs were labeled with fluorescent dyes as described above. For noncovalent postlabeling, nanoparticle solutions in PBS were mixed with a solution of Bodipy630 in PBS (Molecular Probes, D10000, NHS deactivated overnight in PBS). FCS allowed quantification of molar nanoparticle concentrations at known anionic polymer concentrations obtained from Farndale and TB microassays (see above). To allow simple conversion between polymer concentrations in μg/mL and molar nanoparticle concentrations, using these two techniques, we estimated molecular weights of the anionic fraction of nanoparticles with respect to the corresponding reference polymer of Farndale and TB microassays, yielding 0.61 MDa, 1.30 MDa, and 2.37 MDa for PAA, AMSA, and AMBS nanoparticles, respectively, and 2.40 MDa for PLNs.

Nonspecific fetal bovine serum (FBS) labeling: 50 μL of FBS (Gibco, 10500-054, lot 08F2381K) was mixed with 0.2 mL of carbonate buffer (0.1 M carbonate, Sigma-Aldrich, 0.15 M NaCl, VWR, pH 8.0), and 0.67 mg of OG488-NHS (Invitrogen, 06149 Oregon Green 488 carboxylic acid, succinimidyl ester, 6-isomer) in 2.5 μL dry DMSO was added, and the mixture shaken at r.t. for 3 h. The labeled FBS (FBS-OG) was purified by SEC using a PD MiniTrap (GE Healthcare) and PD MidiTrap (GE Healthcare) sequentially; both were equilibrated in PBS. The final solution was sterile filtered (0.2 μm), aliquoted, and stored in the freezer (at −20 °C). Before FCS measurements, a 1/20 dilution of the FBS-OG stock was mixed with desired nanoparticle samples in PBS (diluted 1/10 in the diluted FBS-OG solution) and incubated at 37 °C, 300 rpm in a ThermoMixer for the given amounts of time. Data were compared to the FBS control at same time point (one-way ANOVA, Tukey’s multiple comparison test). For stability measurements in 10% (v/v) unlabeled FBS, CF488 covalently modified nanoparticles (see above) in PBS were used and incubated at 37 °C, 300 rpm in a ThermoMixer. Data were compared between ± FBS using one-way ANOVA, Šidák’s multiple comparison test, with preset pairs.

FCS and FCCS measurements were conducted on a commercial LSM 880 (Carl Zeiss, Jena, Germany), and data were analyzed using the PyCorrfit program 1.1.6.^69^ Dilution series of OG488, CF488, or Alexa647 in PBS were used to calibrate the confocal volume, yielding the *x*–*y* dimension of the confocal volume (ω_*xy*_^2^), which was needed to calculate the diffusion coefficients (*D*) of the subsequent samples by plugging in the obtained diffusion times (*τ*_D_) from the autocorrelation analysis:
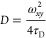


All measurements were performed at 37 °C, while the diffusion coefficients were corrected for the higher temperature used: OG488 (*D* = 5.49 × 10^–6^ cm^2^/s at 37 °C, *D* = 4.1 × 10^–6^ cm^2^/s at 25 °C) and Alexa647 in PBS (*D* = 4.42 × 10^–6^ cm^2^/s at 37 °C, *D* = 3.3 × 10^–6^ cm^2^/s at 25 °C).^70^ 488 and 633 nm excitation light was provided by an Ar^+^ laser and HeNe-laser, respectively. Appropriate filter sets were selected to collect the fluorescence signal and split the two channels sufficiently (for FCCS). The laser beams were focused (200 μm above the glass plate) through a 40× C-Apochromat water immersion objective (NA 1.2) into a sample droplet of 5 μL that was placed onto an ibidi eight-well plate (80827, ibidi, Germany). For each sample, 25 × 5 s intensity traces were recorded, auto-, and cross-correlated. Auto- and cross-correlation curves shown in the figures are always the average curves of the entire measurement of 125 s each. The following one-component fit (*G*_1comp_ (τ)) was used for one-component data, while *G*_2comp_ (τ) was used for two-component systems:
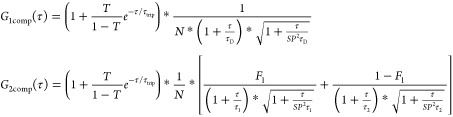
*τ*_D_ is the diffusion time, while τ_1_ and τ_2_ are diffusion times of corresponding fractions *F*_1_ and *F*_2_; *τ*_trip_ is the triplet time (fixed between 1–10 μs) of the triplet fraction *T*; *N* = *n*1 + *n*2 is the effective number of diffusing particles in the confocal volume, and SP is the structural parameter defined as the ratio of height to width of the confocal volume (fixed to 5). The Einstein–Stokes equation was subsequently used to calculate hydrodynamic radii (*R*_h_) via the obtained diffusion coefficients (*D*). For the study with FBS-OG, free FBS-OG was first measured alone to find the corresponding *τ*_D_, which was subsequently fixed as τ_1_, while τ_2_ was fixed to a diffusion time corresponding to the hydrodynamic size of the particles used in the measurement. The data of this FBS-OG incubation (when mixed with unlabeled nanoparticles/PLNs) were subsequently fitted using *G*_2comp_ (τ) with the two fixed diffusion times τ_1_ and τ_2_ to yield the corresponding fractions *F*_1_ and *F*_2_. *F*_2_ was then plotted in the figures as the particle fraction.

A standard FCCS control sample was measured to define the maximum cross-correlation amplitude (FCCS Standard, IBA Sciences, 5-0000-504). The relative cross-correlation amplitude θ is calculated by^71^
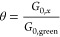
where *G*_0,green_ is the autocorrelation amplitude of the green channel at τ = 0, while *G*_0,*x*_ is the cross-correlation amplitude at τ = 0.

### SPARTA

SPARTA is a previously reported method for label-free, high-throughput analysis of single nanoparticles by Raman spectroscopy that affords single-particle detail at the population level.^40^ A custom confocal Raman microspectroscope was used for SPARTA measurements. Onto a Cerna platform (Thorlabs, UK) was assembled a spectrograph (HoloSpec-F/1.8-NIR, Andor, UK) coupled with an iDus 416ALDC-DD (Andor, UK) thermoelectrically cooled (−60 °C) backilluminated CCD camera. A 63×/1.0 NA WI objective (W Plan-Aprochromat, Zeiss, Oberkochen, Germany) was immersed in sample solutions, and particles were optically trapped with concurrent Raman excitation using a 785 nm laser (200 mW, Cheetah, Sacher Laser Technik, Germany). A 20 s exposure was used for each trapped particle, before the laser was disabled for 1 s to allow release of the trapped particle and a new particle to diffuse into the confocal volume. Blank Gibco DPBS (ThermoFisher Scientific) was measured at 20 s exposure for background subtraction. Raman spectra were analyzed using custom MATLAB scripts for cosmic spike removal, spectral response correction (785 nm reference standard National Institute of Standards and Technology, US), background subtraction, baseline correction, smoothing, and normalization.

### SANS

Samples were prepared as described previously yielding a final theoretical POPC concentration of 1.5 mg/mL after purification. Buffer exchange for SANS measurements was then performed using deuterated PBS. Gibco PBS tablets (ThermoFisher Scientific) were dissolved in D_2_O to obtain deuterated PBS. A PEGonly and a PEG-0.3-film sample in PBS were passed through a PD MidiTrap column (GE Healthcare) equilibrated in deuterated PBS, yielding a final concentration of 1.0 mg/mL. All the measurements were performed at the ZOOM beamline of the ISIS pulsed neutron source at the Rutherford Appleton Laboratory, Didcot, UK. A sample changer and 2 mm path length quartz cuvette cells were used. The beamline was configured with L1 = L2 = 4 m, where L1 is the source to sample distance, and L2 is the sample to detector distance, yielding a scattering variable (*Q*) range of 0.001 to 1 Å^–1^. Samples were measured for 15 μAmps (SANS) and 5 μAmps (TRANS). SANS data were reduced with MantidPlot.^85^ SasView v5.0.3 was employed to fit the experimental data using a core–shell ellipsoid and a core–multishell fit for PEGonly and PEG-0.3-film, respectively. Fits were performed over a *q* range of 0.00416 < *q* < 0.84204 Å^–1^ with a distribution of 0.2 applied to the equatorial core radius in the Core–Shell Ellipsoid model and a distribution of 0.3 applied to the radius in the Core Multi Shell model (see fit parameters Tables S1 and S2).

### Anticoagulation Assays

Antifactor Xa tests (Iduron, Anti-Xa Heparin XAE-200) were conducted according to the manufacturer’s instructions, except the assay was scaled down to lower volumes. Twenty-five micrograms of Factor Xa (EXA-25) and 5 IU antithrombin (PAT-5) were each suspended in 10 mL of Tris buffer (0.05 M Trizma Base, 0.175 M NaCl, 0.1% (w/v) PEG6000, 0.0075 M EDTA, pH 8.4). Five milligrams of Xa substrate (SXE-5.0) was suspended in 10 mL of ddH_2_O. Standard series of heparin (Sigma H3393, 189 USP/mg) was prepared in PBS. A Thermomixer set to static at 37 °C was used, and reagents plus samples were mixed in protein low-bind 1.5 mL Eppendorf tubes. All data points represent duplicates of two separately pipetted tubes. Ten microliters of sample was mixed with 40 μL of Tris buffer and equilibrated at 37 °C for 2 min. A total of 50 μL of antithrombin, 50 μL of Factor Xa, 50 μL of Xa substrate (all from above), and finally 50 μL of 20% (v/v) acetic acid were added to the sample sequentially, with 2 min equilibration at 37 °C for each. Controls included PBS instead of the sample, addition of all of the reagents the wrong way around, and spiking the nanoparticle samples with known amounts of heparin. The final 300 μL solutions for each sample was pipetted into a transparent 96 flat bottom well plate and absorbance read on a plate reader (SpectraMax M5, Molecular Devices) from 350–500 nm; absorbance at 405 nm was used for the calculations. Some nanoparticle samples gave an elevated baseline due to light scattering, which was corrected by subtracting the control sample (everything added the wrong way around) or by subtracting an exponential fit that follows the scattering curve.

### HepG2 Viability Assays

HepG2 cells were cultured using a collagen I coated flask (1 μg/cm^2^ collagen I, A10483-01, Thermofisher Scientific). Culture medium was composed of 500 mL of DMEM (Sigma, D6546), 50 mL of fetal bovine serum (FBS, Gibco), 5 mL of l-glutamine (Sigma, G7513) and 5 mL of P/S (Sigma, P4333). A LIVE/DEAD assay (Thermo Fisher Scientific) was performed following manufacturer instructions using a 96-well plate format. Twenty-four h before seeding cells, 96-well plates were coated with 1 μg/cm^2^ collagen I. A total of 100 μL of 250 000 cells/mL (yielding 25 000 cells/well) in culture medium were seeded in each well and incubated for 24 h at 37 °C. The next day, spent medium was replaced with 90 μL of fresh culture medium and 10 μL samples in PBS or PBS (control). The plates were subsequently incubated for another 24 h. As positive (all dead cells) controls, 10 μL of a 10 mg/mL saponin (47036-50G-F, BioChemika) solution in PBS was added just before the LIVE/DEAD assay readout, and cells were incubated for 10 min. Ten microliters of calcein AM and 20 μL of EthD-1 in 10 mL of PBS were mixed to yield the LIVE/DEAD reagent. After the wells were washed with 3 × 100 μL of PBS, 100 μL of this reagent solution was added to each well. Plates were then incubated for about 45 min in the dark at r.t. Fluorescence was subsequently read on a plate reader (SpectraMax M5, Molecular Devices), measuring nine points across each well and using the average values for calculating % viability compared to PBS controls.

### RAW Cell Viability and Cell Association Assays

Cytocompatibility of nanomaterials developed in this study was studied according to the standard procedure BS ISO 19007:2018.^72^ RAW 264.7 cells were cultured in DMEM medium (high glucose) containing fetal bovine serum (FBS, Gibco, 10% (v/v)) and P/S (1% (v/v), Sigma-Aldrich). Briefly, 15 000 RAW 264.7 cells/well were seeded in the wells of a 96-well plate according to the standard plate setup of BS ISO 19007:2018. Plates were incubated at 37 °C for 24 h, before the spent medium was replaced with 180 μL of fresh medium and 20 μL of nanomaterial solution in PBS or controls (PBS as negative control and aminated PS beads (Sigma-Aldrich, L9904) as positive control). The plates were subsequently incubated for another 24 h at 37 °C. After the incubation, the supernatant was removed, and 120 μL of a mixture of MTS (317 μg/mL, Abcam, ab223881) and PMS (7.3 μg/mL, Sigma-Aldrich, P9625) in phenol-red free RPMI medium was added per well before measuring the absorbance at 490 nm using a plate reader (SpectraMax M5, Molecular Devices) after 1–2 h incubation at 37 °C in the dark.

For cell association experiments, DiD-labeled vesicle samples were first matched to the same fluorescence by measuring the stocks using a plate reader (SpectraMax M5, Molecular Devices) and diluting with PBS. 3 × 10^5^ RAW 264.7 cells were seeded per well in a 24-well plate and incubated overnight. The next day the supernatant was removed and replaced with a mixture of 375 μL of full medium (DMEM + 10% (v/v) FBS + 1% (v/v) PS) with 125 μL of PBS or vesicle sample in PBS. Cells were incubated at 37 °C for 2 h before scratching the cells from the well bottoms and washing three times with PBS. Samples were then measured using a flow cytometer (BD LSRFortessa I) keeping the settings constant for all of the measurements. Media fluorescence values were exported from the software, and % of cell association was calculated by setting the NoPEG sample to 100% and normalizing all the data to this sample. Because of the high variation between the independent experiments, the plots for the three experiments are given separately.

### Zebrafish Embryo Experiments

Experiments involving zebrafish were conducted in accordance with UK Home Office requirements (Animals Scientific Procedures Act 1986, project license P5D71E9B0). Transparent TraNac mutant fish were obtained from Julian Lewis, London Research Institute, London. Fish were kept in the CBS facility of Imperial College London and were reared and maintained according to standard practices at 28.5 °C on a 14-h light/10-h dark cycle. Embryos were raised in E2 water supplemented with 0.3 ppm methylene blue.^73^ Embryos were kept in Petri dishes at a density of ∼50 embryos per dish, and E2 water was replaced daily.

At 3 days postfertilization (dpf), live zebrafish embryos were anesthetized in a solution of 4.2% (w/v) MS-222 and mounted on a 2% (w/v) agarose gel injection plate. The embryos were then injected with 0.5 nL of nanoparticle solution into the caudal vein using borosilicate capillaries (outer diameter 1.0 mm, inner diameter 0.78 mm, length 100 mm; Harvard, Apparatus, Holliston, MA, USA) in a Flaming/Brown P-97 micropipette-puller (Sutter, Novato, CA, USA) with the settings: heat 855, pull 150, velocity 80, and time 94. The injections were performed using a Narishige IM300 microinjection pressure controller (Narishige-group Tokyo, Japan). The injection volume was controlled using an eyepiece reticule (NE120, Pyser-SGI, Edenbridge, UK), and the needle was controlled using a micromanipulator (M3301 Micromanipulator Right hand World Precision Instruments Ltd. Hitchin, UK) and stereomicroscope (Nikon SMZ-1000).

After injection, the embryos were tracked over time using a Leica stereomicroscope (Leica M165 C) with 2.0× objective (Leica), a Leica EL6000 external light source, and Leica DFC7000 T camera. Ten second videos were captured using a GFP filter, 2 × 2 binning, 10× gain, and 8× magnification. Immediately after imaging, the embryos were put back into fresh E2 water, transferred to the incubator, and kept at 28.5 °C until the end of the experiment.

Nanoparticle circulation was analyzed using customized Fiji macros (available on request). Nanoparticle circulation was analyzed within a region of interest (1200 × 450 μm) covering the tail region of the zebrafish embryos. The circulating NP fraction was identified by detecting all fluorescent signals that changed between the different frames in the time series. This was done by subtracting each individual frame of the video by its subsequent frame. This image series was then merged into a single maximum projection. The circulating areas in the maximum projection were then detected using a Fiji watershed plug-in (Watershed on gray level images (http://bigwww.epfl.ch/) 02.2008 Biomedical Imaging Group (BIG), EPFL Lausanne, Switzerland) with the following settings: Gaussian Blur radius: 2 pixels; 4-connected, Min/Max 0–110. The total circulating area was then measured using the built-in “analyze particle” plug-in available in Fiji. All measurements were normalized to the total fluorescence detected in the ROI yielding the circulation fraction plotted in the graphs. The total fluorescence was detected using the exact same procedure as outlined for the circulation fraction but without the initial subtraction step. Because of the sensitivity of this method to any movement in the video, some videos of a total of 127 had to be excluded from the analysis. These were videos of embryos that visibly moved during imaging (32/127), any videos of unhealthy embryos (10/127), any analysis that yielded an erroneous circulation fraction >1 (7/127), which were all excluded from the analysis. Sufficient numbers of embryos (4–18) were used for each condition yielding enough videos per condition that fit the above criteria.

### Vero Cell Viability Assay

African green monkey kidney (Vero) cells (Nuvonis Technologies) were maintained in OptiPRO SFM (Life Technologies) containing 2× Glutamax (Gibco) and seeded into 96-well so as to be confluent the following day. A two-fold serial dilution of samples was performed as in the SARS-CoV-2 inhibition assay, but OptiPRO SFM, 2× Glutamax was added instead of virus, and the plate was incubated for 1 h at 37 °C, 5% CO_2_. Diluted samples were transferred to the cell plate and incubated for 1 h at 37 °C, 5% CO_2_ before washing twice with OptiPRO SFM, 2× Glutamax and incubating for 42 h. Cell viability assay was performed to the manufacturer’s instructions, the same as for RAW 264.7 cells as described above. In short, MTS and PMS cell viability reagents were prepared and added to cells, incubated for 1 h at 37 °C, 5% CO_2_ and absorbance measured at 490 nm.

### HSV-2 Inhibition Assays

Vero Cells (ATCC-CCL81) and HSV-2 (kindly gifted from Prof. M. Pistello, University of Pisa) propagated in Vero Cells were used. Cells were cultured in DMEM medium (Gibco) supplemented with 10% (v/v) FBS (Gibco) and 1% (v/v) P/S (Gibco). For dose–response, Vero cells were plated 24 h before the experiment (90 000 cells/well in a 24 well plate) in order to have a confluent monolayer on the day of the experiment. A sample of interest was serially diluted in DMEM (2% (v/v) FBS, 1% (v/v) P/S) and incubated for 1 h at 37 °C and 5% CO_2_ with a fixed amount of virus (MOI, 0.005 PFU/cell). A control was prepared with no compound. The mixture was then added onto cells (200 μL per well) and incubated for 1 h at 37 °C and 5% CO_2_. The inoculum was then removed, and cells were overlaid with methyl-cellulose rich (0.45% w/v) DMEM supplemented with 2% (v/v) FBS, 1% (v/v) P/S. Cells were then incubated at 37 °C and 5% CO_2_. Twenty-four hours post infection, supernatant was removed, and cells were stained with crystal violet. Plaques were then manually counted through optical microscopy. The percentage of infectivity was calculated dividing the number of plaques at a given concentration by the number of plaques present in the untreated control. The concentration at which 50% of the viruses are inhibited (EC_50_) was calculated in Prism 9.

For virucidal assays, Vero cells were plated 24 h before the experiment (14 500 cells/well in a 96 well plate) in order to have a confluent monolayer on the day of the experiment. The compound of interest was mixed at a certain concentration with a fixed amount of virus (∼10^5^ PFU/mL) in DMEM (2% (v/v) FBS, 1% (v/v) P/S) and incubated for 1 h at 37 °C and 5% CO_2._ A control was prepared with no compound. The solution was then serially diluted, and the different dilutions were added onto cells (100 μL per well) and incubated for 1 h at 37 °C and 5% CO_2_. The inoculum was then removed, and cells were overlaid with methyl-cellulose rich (0.45% w/v) DMEM supplemented with 2% (v/v) FBS, 1% (v/v) P/S. Cells were then incubated at 37 °C and 5% CO_2_. Twenty-four hours post infection, the supernatant was removed, and cells were stained with crystal violet. Plaques were then manually counted through an optical microscope. Viral titer was then calculated.

### SARS-CoV-2 Inhibition Assay

African green monkey kidney (Vero) cells (Nuvonis Technologies) were maintained in OptiPRO SFM (Life Technologies) containing 2× GlutaMAX (Gibco) and seeded into 96 wells so as to be confluent the following day. In a separate 96-well dilution plate, reference samples (heparin sodium salt from porcine intestinal mucosa, Sigma H3393, unfractionated heparin (UFH), 18 kDa, three different lots used), UFH (TCI Chemicals, H0393), UFH (Millipore, 375095), and WHO Reference Panel: anti-SARS-CoV-2 immunoglobulins, NIBSC code: 20/150^56^) or test samples were two-fold serially diluted in duplicate in OptiPRO SFM, 2× Glutamax before the addition of 100 TCID_50_/well of SARS-CoV-2 and incubation for 1 h at 37 °C, 5% CO_2_. The viruses used were B.1 lineage hCoV-19/England/IC19/2020 (GISAID accession ID: EPI_ISL_475572, a WT D614G isolate) and B.1.351 lineage hCoV-19/England/205280030/2020 (EPI_ISL_770441). Samples were then transferred from the dilution plate to the plate containing Vero cells and incubated for 1 h at 37 °C, 5% CO_2_. The inoculum was then removed, the cells were washed twice with OptiPRO SFM, 2× Glutamax, the medium was replaced, and plates were returned to 37 °C, 5% CO_2_ for a further 42 h before fixing cells with 4% (v/v) PFA. Plates were washed twice with PBS before incubation with methanol, 0.6% (v/v) H_2_O_2_ at RT for 20 min. A 1:3000 dilution of 40143-R019 rabbit mAb to SARS-CoV-2 nucleocapsid protein (Sino Biological) in PBS, 5% (w/v) milk powder was added to plates and incubated for 1 h at RT. Four PBS washes were performed before adding a 1:3000 dilution of sheep antirabbit HRP conjugate (Sigma) in PBS, 5% (w/v) milk powder, 1% (w/v) BSA, and incubating for 1 h at RT. Plates were washed four times with PBS. TMB substrate (Europa Bioproducts) was added and developed for 20 min before stopping the reaction with 1 M HCl. Plates were read on a spectrophotometer (SpectraMax M5, Molecular Devices), and the OD at 620 nm was subtracted from the OD at 450 nm. The infectivity of virus in the presence of different concentrations of inhibitory sample was calculated as a percentage of the control.

### Malaria Assays

*Plasmodium falciparum* strains 3D7, D10, and W2mef were cultured in human O^+^ RBCs as described elsewhere^74^ using RPMI-HEPES (Sigma-Aldrich, R5886) medium supplemented with 5g/L Albumax II (Gibco),^75^ 0.292 g/L l-glutamine, 0.05 g/L hypoxanthine, and 0.025 g/L gentamicin. 5% (w/v) sorbitol was used for synchronization.^76^*Plasmodium knowlesi* strain A1-H.1 parasites were cultured in human O^+^ RBCs as published elsewhere.^77^ The culture medium consisted of RPMI-HEPES medium supplemented with 2 g/L dextrose, 0.292 g/L l-glutamine, 2.3 g/L sodium bicarbonate, 0.025 g/L gentamicin, 0.05 g/L hypoxanthine, 5 g/L Albumax II (Gibco), and 10% (v/v) equine serum (Life Technologies). All parasites were cultured at 37 °C with a gas mixture of 90% N_2_, 5% O_2_, 5% CO_2_.

Suspension culture growth inhibition assays were performed as described elsewhere with some alterations.^21^ Briefly, 135 μL of parasite mix at 5% hematocrit and 1–2% parasitemia (*P. falciparum* was synchronized prior to the assay to start the assay with a synchronous trophozoite/schizont culture) in the corresponding culture media was mixed with 15 μL of PBS or test samples in flat bottom 48-well plates. Plates were placed stacked on each other (using only the top two plates for each stack) and surrounded with wet tissue paper in a gastight plastic box. The box was gassed with the above mixture and incubated at a tilt angle of about 15° using a shaker at 185 rpm inside a cell culture incubator. After an overnight (typically 18–24 h) incubation, 10 μL of each well suspension was transferred to a U-bottom 96-well plate (each well contains 200 μL PBS), before spinning down the plate, discarding the supernatant, and adding 200 μL of the staining solution (1/5000 dilution of SYBR Green (Invitrogen, S7563) in PBS). After 20 min of staining, the plates were washed three times with 200 μL of PBS and run on a flow cytometer (platereader, BD LSRFortessa II). EC50-curves were analyzed using QtiPlot (https://www.qtiplot.com/download.html).

### IFA and Microscopy

IFA protocol: a synchronous *P. falciparum* 3D7 culture at 1% hematocrit and 10% parasitemia (schizonts) in parasite culture medium was incubated together with a nanoparticle solution (covalently labeled with Cy5/CF488), which was diluted in the parasite mix by 1/10. After gassing the 15 mL Falcon tubes with the above gas mixture, the samples were incubated at 37 °C for about 8 h. The sample was then run through a MACS column (CS columns 130-041-305 from Miltenyi Biotec) to remove remaining iRBCs and free hemozoin. The washthrough (RBCs and free merozoites) was collected and fixed by mixing 1 to 1 with 4% (v/v) paraformaldehyde and 0.4% (v/v) glutaraldehyde in phosphate buffer (0.1 M phosphate, 0.05 M NaCl) for 20 min. This was followed by 20 kRCF centrifugation and two PBS washes before blocking in 3% (w/v) BSA/PBS overnight at 4 °C. The next day, a 1/250 dilution of primary antibody (rabbit anti-MSP1)^78^ in 3% (w/v) BSA/PBS was added and incubated at r.t. rotating for 45 min, followed by 3 × 10 min washes in PBS and incubation with 1/500 dilution of goat antirabbit antibody-Alexa488 (Invitrogen, A11008) or antirabbit antibody-Alexa647 (Invitrogen, A21245) in 3% (w/v) BSA/PBS for 30 min (rotating) before washing 3 × 10 min with PBS. 4′,6-Diamidino-2-phenylindole (DAPI ) was diluted 1:4000 in PBS to stain the parasite nucleus. Every centrifugation step was performed at 20 kRCF (5 min). Samples were mounted on objective glass using Vectashield for widefield microscopy. For STORM, samples were put in ibidi eight-well chambers that were previously coated for 2 h with 10 mg/mL protamine (Sigma-Aldrich, 80827) in water and washed with PBS. The samples were allowed to sediment and attach to the protamine surface overnight at 4 °C.

Images (z-stacks) were recorded on a Nikon Ti Microscope with a 100× oil immersion objective. EpiDEMIC plug-in in Icy (50 iterations) was used to deconvolve the z-stacks.^79^ Images were subsequently processed in Fiji.

For live videoing, a synchronous *P. falciparum* 3D7 culture was Percoll purified to extract late stages, which were subsequently incubated in complete parasite culture medium and including compound 2 (C2)^80^ at 2 μM to inhibit egress. Just before imaging, C2 was washed away by centrifugation and resuspension in complete parasite culture medium. A mixture of this schizont mix, fresh RBCs, and Cy5-labeled AMSA nanomimics was incubated in complete parasite culture medium using ibidi glass bottom plates and imaged live at 37 °C on a Nikon Ti microscope with a 100× oil immersion objective.

### STORM Imaging

Prior to imaging, TetraSpeck microspheres (100 nm in diameter; Thermo Fisher Scientific) were introduced to the samples to serve as fiducial markers. They were diluted 1:400 (v/v) with DPBS (Gibco, Thermo Fisher Scientific) and incubated for 20 min at room temperature, followed by three DPBS washes. The samples were then soaked in imaging buffer with the following composition: Tris buffer (160 mM Tris, 40 mM NaCl, pH adjusted to 8.0), 10% (w/v) glucose, 0.5 mg/mL glucose oxidase from *Aspergillus niger* (G7141), 47 μg/mL catalase from bovine liver (C1345), and 10 mM cysteamine (pH adjusted to 8.0). All of the buffer components were purchased from Sigma-Aldrich. To minimize oxygen entry, the sample slide was sealed with parafilm. STORM imaging was conducted with a Nikon Ti Eclipse inverted microscope (Nikon, Tokyo Japan), with cube filters (excitation: Chroma ZET405/488/561/640x, emission: Chroma ZET405/488/561/640m) and TIRF dichroic ZET405/488/561/640bs, and equipped with Cairn laser module (Cairn Research, Kent, UK) with 300 mW 405 nm, 200 mW 488 nm, and 140 mW 642 nm lasers used here. A CFI SR Apo TIRF 100× oil objective (N.A. 1.49) was used with a 1.5× Optovar lens, resulting in a 150× magnification. The pixel size of the camera (Andor iXON Ultra 888 EMCCD, Oxford Instruments, Belfast, UK) was 13 μm. The image acquisition was controlled with MetaMorph and Micro-Manager open-source software. A 128 × 128 pixels region of interest was imaged, and a diffraction-limited image was acquired for reference before starting the STORM acquisition. 30 000 frames, with an exposure time of 30 ms/frame, 100% laser power, and electron multiplying gain of 300, were recorded for each image. The acquisition was started only when an optimal level of fluorophore photoswitching was reached. The channels were recorded sequentially starting from the 488 nm channel and followed by the 642 nm channel. The 405 nm channel (DAPI) was only imaged in a diffraction-limited mode.

The image stacks were reconstructed with the ThunderSTORM plugin in Fiji.^81^ The following reconstruction parameters were used:

#### Image Filtering

Filter: Difference-of-Gaussians filter (Sigma1 = 1.0 px, Sigma2 = 1.6 px)

#### Approximate Localization of Molecules

Method: Local maximum

Peak intensity threshold: std(Wave.F1)

Connectivity: 8-neighborhood

#### Subpixel Localization of Molecules

Method: PSF: Integrated Gaussian

Fitting radius (px): 3

Fitting method: Weighted least-squares

Initial sigma (px): 1.6

Multiemitter fitting analysis: enabled

Maximum of molecules per fitting region: 3

Model selection threshold (p-value): 1.0 × 10^–6^

The intensity range (photons) not limited.

Regarding image postprocessing, in addition to drift correction conducted with the ThunderSTORM cross-correlation algorithm, sigma-based filtering was conducted to remove the noise/background in the lower end and signal from partially overlapping fluorophores in the upper end. The lower and upper sigma filtering values were the following: 647 nm channel [90, 215], 488 nm channel [70, 165]. Moreover, a chromatic aberration correction was conducted to the images using Detection of Molecules (DoM) plugin in Fiji,^82^ using a correction mask created with the same plugin from diffraction-limited images of densely arranged fiducial markers only. To visualize the data, a Normalized Gaussian method was used, with a magnification of 10 and an uncertainty value calculated image-specifically. The channels were aligned manually in Fiji using the fiducial markers as reference points.

### Mouse Experiments

Animal works in this study were carried out according to the Animals (Scientific Procedures) Act 1986 Amendment Regulations 2012 (SI 2012/3039) and were approved by Imperial College London Ethical Review Committee (PPL and PDA3EBA4A). Mice were kept in individually ventilated cages.

For the initial toxicity studies (histopathology evaluation), naïve BALB/c mice were first placed in a 37 °C heat-box for 10 min before i.v. injection of 100 μL of a sterile nanoparticle solution in PBS at concentrations to yield a dose of 1.5 mg/kg active polymer (anionic polymer amount quantified by Farndale microassays, see above) or 100 μL PBS (control). Mice were euthanized 4 or 9 days post injection and heart, kidney, liver, lung, and spleen tissues were collected and fixed in neutral buffered formalin (10% (w/v), Sigma-Aldrich, HT5014–1CS) at RT for 24 h, during which formalin was changed once. Tissues were subsequently washed and stored in 70% (v/v) ethanol. Tissues were paraffin embedded, sectioned (approximately 4 μm thick), and stained with hematoxylin and eosin (H&E) and then examined and compared to the PBS control. Images were taken on a Nikon Ti-E inverted widefield microscope at x20 and x40 objective.

For the *in vivo* parasite experiments, the transgenic *P. berghei* ANKA line Pb2257cl2 (*Pb*ANKA-*Pf*CSP(r)_*PbCSP*_*;* RMgm-4110) was used, expressing GFP-luciferase and *Pf*CSP under the control of *CSP* promoter.^83^ Infections were started by intraperitoneal (i.p.) injection of freshly thawed cryopreserved parasitized RBCs into naïve BALB/c mice. On day five of infection, at approximately 1–2% parasitemia, fresh parasitized blood was collected by a syringe coated in heparin (300 μg/mL stock, Sigma H3393) by cardiac puncture under nonrecovery anesthesia. Fresh parasitized blood was subsequently injected by i.p. into phenylhydrazine-treated BALB/c mice.

Infected blood for culture was later harvested as above once parasitemia had reached over 1% and cultured as described elsewhere.^65^ Briefly, harvested infected blood was immediately transferred to a Falcon tube with 0.3 mL of heparin stock in PBS and 5 mL of full culture medium: 400 mL of RPMI-HEPES (Sigma-Aldrich, R5886) medium supplemented with 100 mL of FBS (20% (v/v), Gibco), 5 mL of l-glutamine (Sigma, G7513), and 5 mL of Pen/Strep (Sigma, P4333). The sample was immediately spun down at 450*g* for 8 min, the supernatant was removed, and the pellet was resuspended in 20 mL of full culture medium. The culture was split in two, transferred to T75 flasks, and complemented with 20 mL of full culture medium each. Flasks were gassed with a gas mixture of 90% N_2_, 5% O_2_, and 5% CO_2_ and maintained at 37 °C under a gentle shaking condition (60 rpm) until the next morning.

The next day, thin blood smears were used to monitor stage of parasites and when more than 50% of parasites reached the schizont stage, the culture was spun down at 450*g* for 8 min, the supernatant was removed, and the pellet was resuspended in about 5 mL of full culture medium. Late-stage parasites were then extracted from the culture using a MACS column (CS columns 130-041-305 from Miltenyi Biotec) in full culture medium. Concentration of purified parasites was measured using a hematocytometer. Immediately before injections, the culture was spun down at 450*g* for 8 min, the supernatant was removed, and the pellet was resuspended in incomplete culture medium (only RPMI-HEPES and l-glutamine) to a concentration of 2 million parasites per mL. Just before loading into the syringe, this parasite mixture was mixed 1 to 1 with either PBS or nanoparticles in PBS (at 600 μg/mL active polymer). A total of 100 μL of this solution was then i.v. injected via the tail vein into naïve BALB/c mice that were first placed in a 37 °C heat-box for 10 min. Mice were randomized before injection and treatments were blinded. This yielded a parasite dose of 1 × 10^5^ late stages and an active anionic polymer concentration of 1.5 mg/kg. From day 2–6, parasitemia was monitored daily by thin blood smears (Giemsa-stained) until 3 days of positive smears were obtained, mice were then euthanized. Ten microscopy images were taken on each smear (random areas where RBC density was a monolayer) using a Nikon Ti-E inverted widefield microscope at 100× objective. Smears were randomized and imaged blinded. The number of RBCs was counted automatically using PlasmoCount,^84^ while the number of infected RBCs was counted manually using Fiji because PlasmoCount has not yet been extended to *P. berghei* detection. Parasitemia (% of infected RBCs) was calculated by dividing the total number of counted infected RBCs by total number of RBCs (around 2000 per smear, 10 images combined). % of PBS control was given as comparison on the day the parasitemia reached above 1% in the PBS control mice.

### Safety Statement

There are no unexpected, new, and/or significant hazards or risks associated with the reported work.

## Supporting Information Available

Supplementary Figures S1–S16: particle characterization and stability analysis (UV–vis spectroscopy, TEM, cryo-TEM, FCS, FCCS, DLS), cytocompatibility and cell association data (RAW, HepG2), anti-Xa assays (UV–vis spectroscopy), additional zebrafish embryo images, virus inhibition data, parasite inhibition data and images, histopathology of mouse organs, and additional *in vivo* data. Supplementary Tables S1–S2: SANS fitting parameters. Supplementary references (PDF)
Supplementary Movies S1–S5: example movies of zebrafish embryo circulation and *in vitro* merozoite inhibition (AVI1, AVI2, AVI3, AVI4, AVI5)

## Supplementary Material

oc1c01368_si_006.pdf

oc1c01368_si_001.avi

oc1c01368_si_002.avi

oc1c01368_si_003.avi

oc1c01368_si_004.avi

oc1c01368_si_005.avi

## Notes

Raw data are available upon request from rdm-enquiries@imperial.ac.uk. Customized Fiji macros are available on request.
