# Supplementary material for: Potent Virustatic Polymer–Lipid Nanomimics Block Viral Entry and Inhibit Malaria Parasites In Vivo
Source: ACS Cent Sci. 2022 May 3;8(9):1238–57. doi: 10.1021/acscentsci.1c01368 (PMC9092191; doi:10.1021/acscentsci.1c01368)
Supplement: Supplementary file 1 — oc1c01368_si_006.pdf [file oc1c01368_si_006.pdf]

Supporting Information

Potent Virustatic Polymer-Lipid Nanomimics Block Viral Entry and Inhibit Malaria Parasites In Vivo

Adrian Najer,[1,2,] Joshua Blight,[2] Catherine B. Ducker,[2] Matteo Gasbarri,[3] Jonathan C. Brown,[4] Junyi Che,[1] Håkon Høgset,[1] Catherine Saunders,[1] Miina Ojansivu,[5] Zixuan Lu,[1] Yiyang Lin,[1] Jonathan Yeow,[1] Omar Rifaie-Graham,[1] Michael Potter,[1] Renée Tonkin,[1] Jelle Penders,[1] James J. Doutch,[6] Athina Georgiadou,[4] Hanna M. G. Barriga,[5] Margaret N. Holme,[5] Aubrey J. Cunnington,[4] Laurence Bugeon,[2] Margaret J. Dallman,[2] Wendy S. Barclay,[4] Francesco Stellacci,[3,7] Jake Baum,[2,*] and Molly M. Stevens[1,5,*]*

1. Department of Materials, Department of Bioengineering, and Institute of Biomedical Engineering, Imperial College London, London, SW7 2AZ, UK.
2. Department of Life Sciences, Imperial College London, London, SW7 2AZ, UK.
3. Institute of Materials, Ecole Polytechnique Fédérale de Lausanne (EPFL), 1015 Lausanne, Switzerland.
4. Department of Infectious Disease, Imperial College London, London, W2 1PG, UK.
5. Department of Medical Biochemistry and Biophysics, Karolinska Institutet, SE-171 77 Stockholm, Sweden.
6. Rutherford Appleton Laboratory, ISIS Neutron and Muon Source, STFC, Didcot OX11 0DE, UK.
7. Institute of Bioengineering, Ecole Polytechnique Fédérale de Lausanne (EPFL), 1015 Lausanne, Switzerland.

Current address: Jake Baum, School of Medical Sciences, University of New South Wales, Sydney, Australia.

* Corresponding authors email: a.najer@imperial.ac.uk, jake.baum@unsw.edu.au, m.stevens@imperial.ac.uk

Supplementary Figures S1-S16

Supplementary Tables S1-S2

Supplementary Movies S1-S5

Supplementary References

Total of 23 pages (S1-S23)

Supplementary Figures

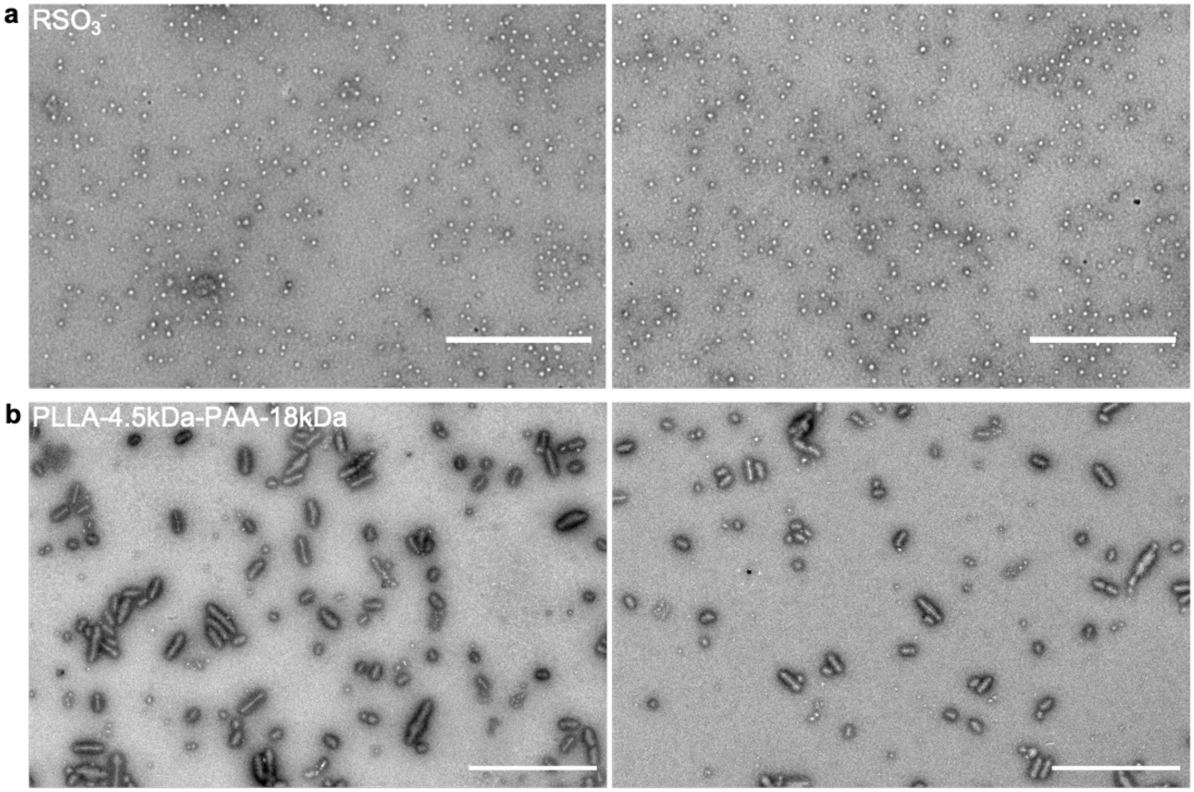

Supplementary Figure 1. Polymer nanomimic morphology. (a) TEM images of polymer nanomimics based on AMSA-modified PDLLA-*b*-PAA. Scale bar, 500 nm. (b) TEM images of polymer nanomimics based on PLLA-*b*-PAA (modified with a mixture of homotaurine (HT) and 3-(Dimethylamino)-1-propylamine (DMAPA)). Scale bar, 500 nm.

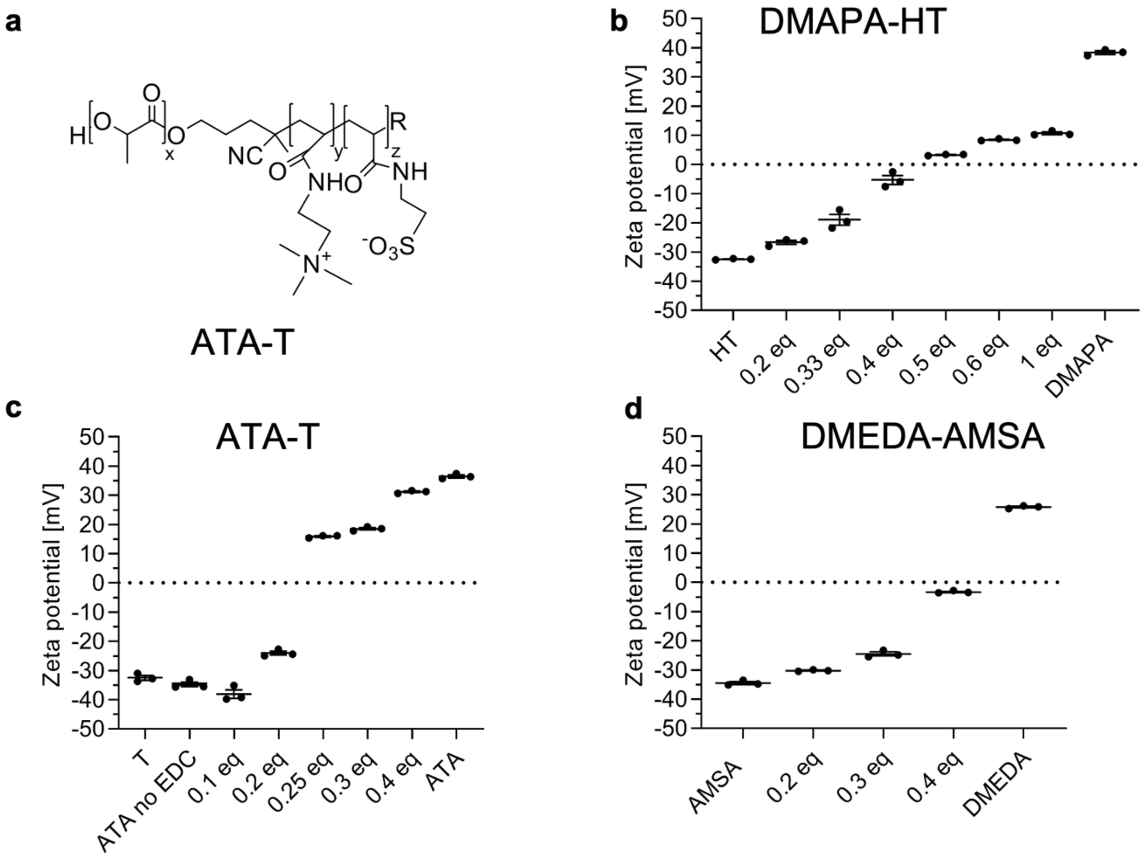

Supplementary Figure 2. Anionic and mixed charge polymer nanoparticle modifications. (a) Chemical structure of a mixed charge example after modification with combinations of (2-Aminoethyl)trimethylammonium (ATA) and taurine (T). (b) Average zeta potential values for a series of HT and DMAPA modifications of nanoparticles (mean ± s.e.m., technical triplicates). (c) Average zeta potential values for a series of T and ATA nanoparticle modifications (mean ± s.e.m., technical triplicates). (d) Average zeta potential values for a series of Aminomethanesulfonic acid (AMSA) and *N,N*-Dimethylethylenediamine (DMEDA) nanoparticle modifications (mean ± s.e.m., technical triplicates).

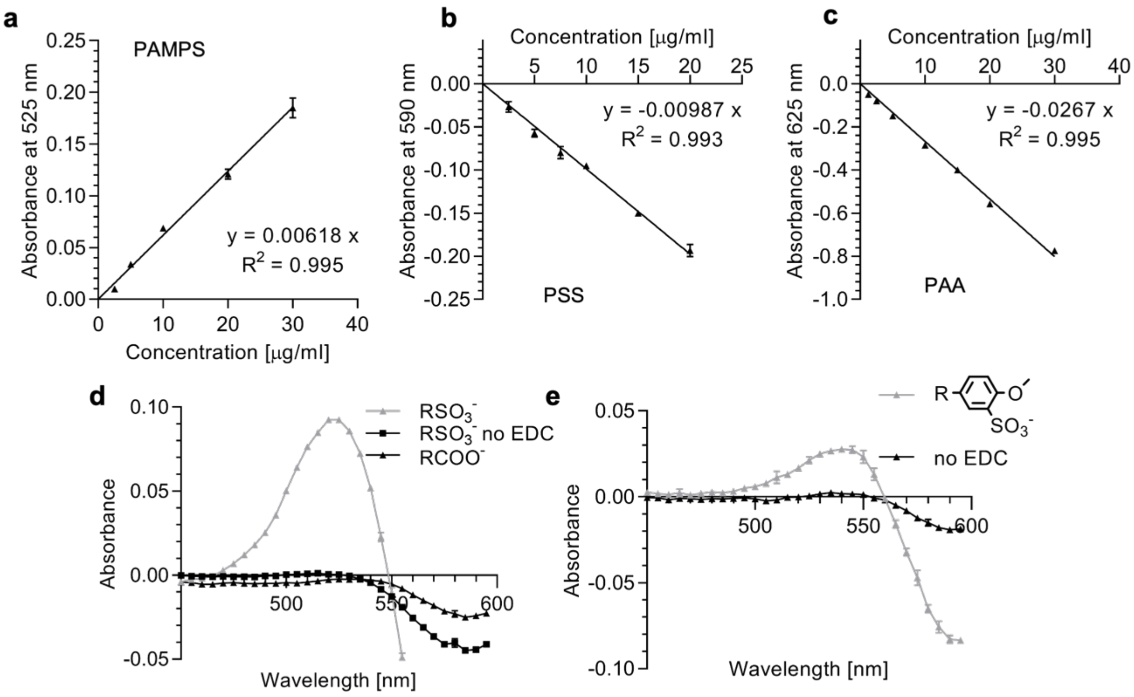

Supplementary Figure 3. Anionic polymer quantification. (a) Calibration curve of a modified Farndale microassay to quantify polysulfonates using Poly(2-acrylamido-2-methyl-1-propanesulfonate (PAMPS, 2 MDa) in PBS as a standard (mean ± s.e.m., technical duplicates). (b) Calibration curve of a modified Farndale microassay to quantify benzene-coupled polysulfonates using Poly(sodium 4-styrenesulfonate) (PSS, 1 MDa) in PBS as a standard (mean ± s.e.m., technical duplicates). (c) Calibration curve of a modified toluidine blue (TB) microassay to quantify polycarboxylates using Poly(acrylic acid) (PAA, 250 kDa) in PBS as a standard (mean ± s.e.m., technical duplicates). (d) Example UV-Vis absorbance curves (after subtraction of DMMB-PBS control) used for quantification of polymer in nanoparticle solutions using Farndale microassay. This shows quantitative detection of polysulfonates on AMSA-modified nanoparticles versus a control (AMSA without EDC) and original PAA nanoparticles (mean ± s.e.m., technical duplicates). (e) Example UV-Vis absorbance curves (after subtraction of DMMB-PBS control) for quantification of polymer in nanoparticle solutions using Farndale microassay showing covalent modification with AMBS via EDC reaction and successful purification from sulfonate reagents (without EDC, also see Supplementary Figure 6a) (mean ± s.e.m., technical duplicates).

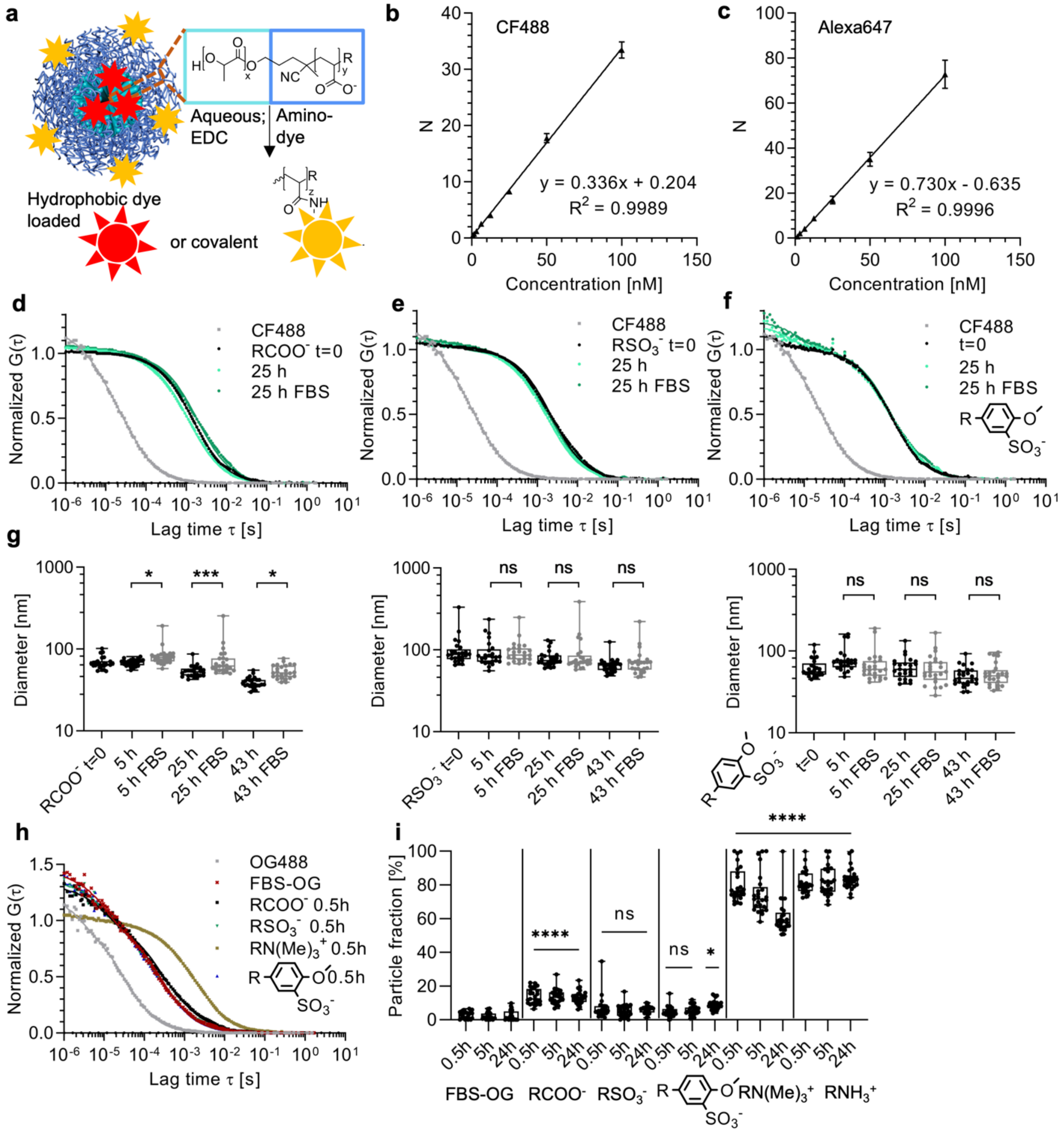

Supplementary Figure 4. FCS study of nanoparticle concentration, stability, and protein fouling. (a) Schematic of fluorescent dye modification of polymer nanoparticles to obtain nanoparticle concentration and size. (b,c) Confocal volume calibration from FCS measurements (*N* = average number of molecules in confocal volume) using known CF488 and Alexa647 concentrations in PBS (mean ± s.d., *n* = 25 (CF488) and 15 (Alexa647) technical replicates, respectively). (d-f) Normalized FCS autocorrelation curves of CF488-labeled PAA, AMSA, and AMBS nanoparticles in PBS or 10% (v/v) FBS at time 0 and after 25 h (average curves of *n* = 25 technical replicates, 5 s each, symbols represent raw data, lines are the fits). (g) Stability of nanomimics over time in +/- 10% (v/v) FBS determined using FCS (*n* = 25 technical replicates, one-way ANOVA with Šidák's multiple comparisons test, $^{*}P < 0.05$, $^{**}P < 0.01$, *ns* = not significant). (h) Normalized FCS autocorrelation curves for mixtures of unlabeled nanoparticles (AMSA, AMBS, ATA modified) with randomly labeled FBS-OG488 at timepoint 0.5 h (raw curves of Figure 1g, average curves

of n = 25 technical replicates, 5 s each, symbols represent raw data, lines are the fits). (i) Non-specific binding of FBS-OG488 to various nanoparticles over time as obtained by two-component FCS fits, repeated experiment of main Figure 1g (n = 25 technical replicates, one-way ANOVA with Tukey's multiple comparisons test, shown comparisons to FBS-OG488 only, ****P < 0.0001, *P < 0.05, *ns* = not significant). Box-plots: Center line, the median; box limits, upper and lower quartiles; whiskers, minimum and maximum values.

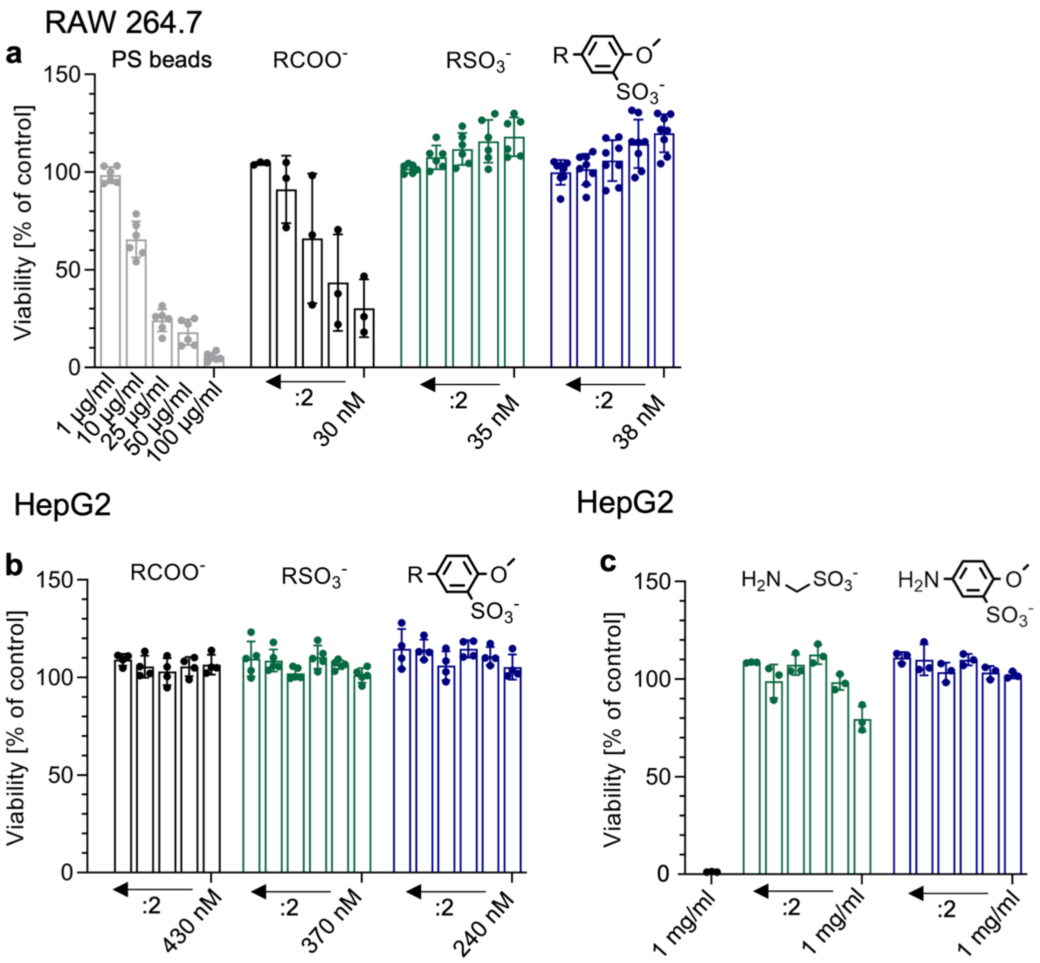

Supplementary Figure 5. Cytocompatibility studies for nanomimics and building blocks. (a) Cytocompatibility of nanomimics tested with RAW 264.7 cell line ($N \geq 3$ independent experiments with technical triplicates). Gray: aminated polystyrene (PS) control particles; black: PAA-based particles with RAFT-endgroup removed (SMES); green: AMSA-modified nanomimics RAFT-endgroup removed (SMES); blue: AMSA-modified nanomimics RAFT-endgroup removed (MUS). (b) Cytocompatibility of nanomimics tested with HepG2 cell line ($N \geq 3$ independent experiments with technical triplicates). (c) Cytocompatibility of building blocks AMSA/AMBS tested with HepG2 cell line ($N = 1$ independent experiment with technical triplicates); black is 0.1% (w/v) saponin. Highest particle concentrations are given with subsequent values corresponding to two-fold serial dilutions.

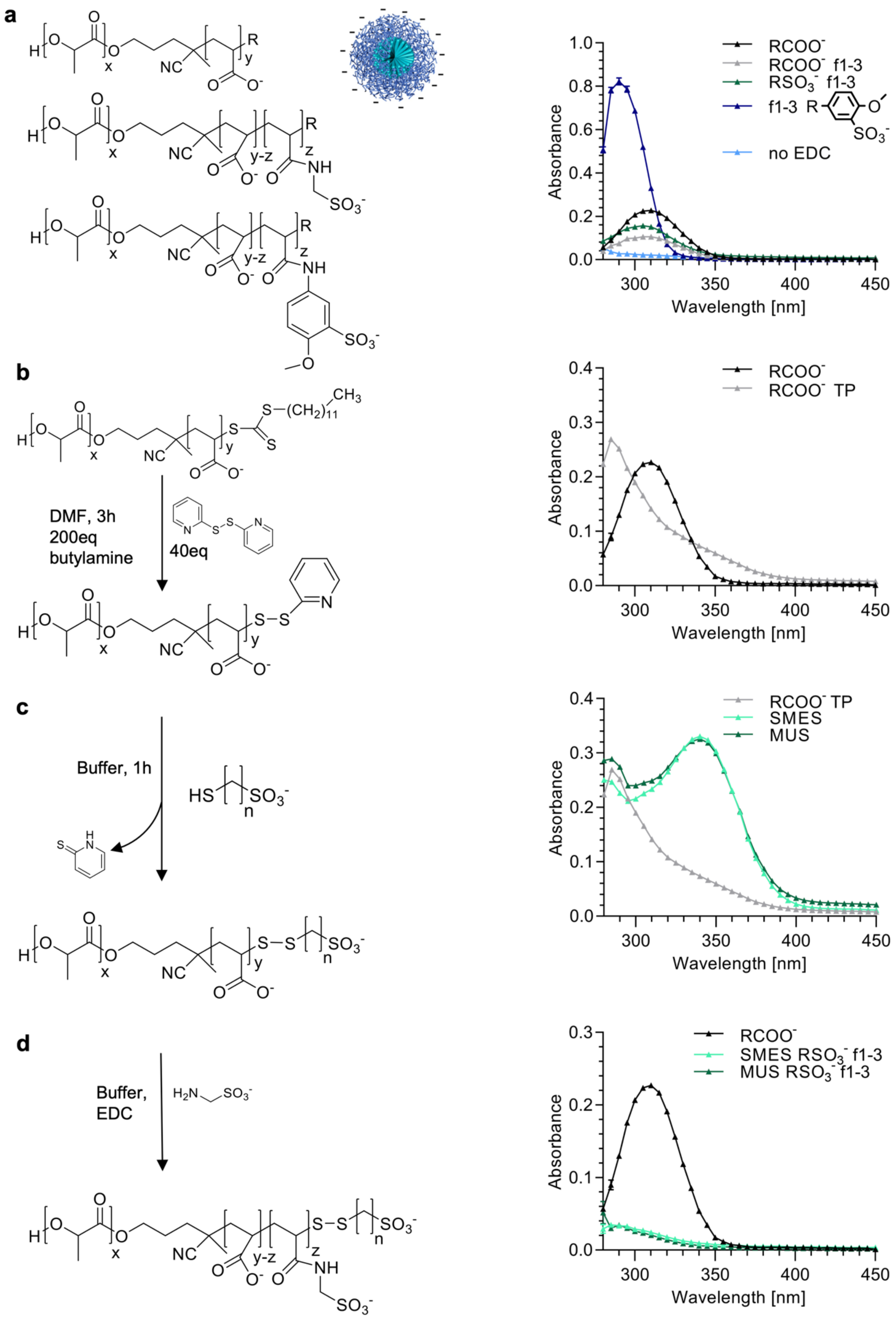
a
b
c
d
DMF, 3h
200eq
butylamine
40eq
Buffer, 1h
Buffer,
EDC
RCOO⁻
RCOO⁻ f1-3
RSO₃⁻ f1-3
f1-3
no EDC
RCOO⁻ TP
SMES
MUS
SMES RSO₃⁻ f1-3
MUS RSO₃⁻ f1-3
Absorbance
Wavelength [nm]

Supplementary Figure 6. Aminolysis and thiol-exchange reaction to remove RAFT-endgroup from copolymer. (a) Schematic of main copolymers used herein and corresponding UV-Vis absorbance after purification showing RAFT-endgroup at 315 nm and AMBS at 290 nm (blue samples 10x diluted compared to others showing complete removal of AMBS when no EDC was used, mean ± s.e.m., technical duplicates). (b) Reaction scheme for aminolysis and thiol protection using DTP and corresponding UV-Vis absorbance of control particles from a) and after RAFT-endgroup removal (n = 1). (c) Schematic of thiol-exchange reaction using SMES (n = 2) or MUS (n = 11) and corresponding UV-Vis absorbance showing appearance of cleaved protection group upon incubation when compared to the protected form after aminolysis from c) (n = 1). (d) Schematic of carboxylic acid modification and final UV-Vis absorbance of nanomimics without RAFT-endgroup in comparison to the control from a) (mean ± s.e.m., technical duplicates).

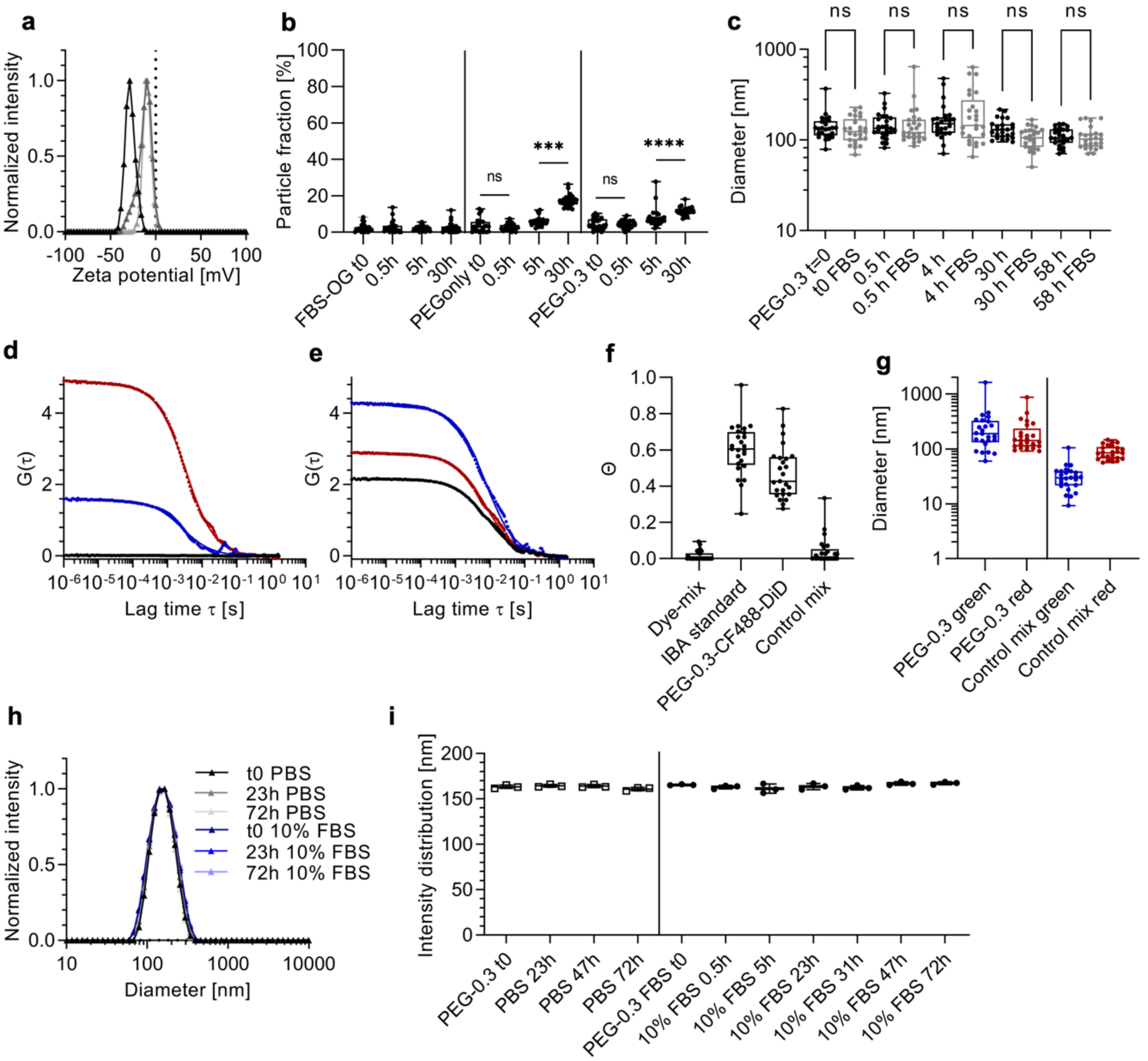

Supplementary Figure 7. PLN morphology characterization and stability study. (a) Zeta potential distribution for NoPEG (black), PEGonly (light gray) and PEG-0.3 PLNs (gray). (b) Non-specific binding of FBS-OG488 to PEGonly or PEG-0.3 PLNs over time as obtained by two-component FCS fits ($n = 25$ technical replicates, one-way ANOVA with Tukey's multiple comparisons test, shown comparisons to FBS-OG488 only, ***$P < 0.001$, ****$P < 0.0001$, *ns* = not significant). (c) Stability of fluorescent PEG-0.3 over time in +/- 10% (v/v) FBS determined using FCS ($n = 25$ technical replicates, one-way ANOVA with Šidák's multiple comparisons test, *ns* = not significant). (d,e) FCCS auto- and cross-correlation curves for green (488 nm excitation, blue), red (633 nm excitation, red), and cross channel (black) for (d) a mixture of PEG-0.3-DiD and PDLLA-AMSA-CF488 nanoparticles; (e) dual labeled PLNs (PEG-0.3-AMSA-CF488-DiD). (f) Relative cross-correlation amplitude (θ) for samples in d,e and standards ($n = 25$ technical replicates, 5 s each). (g) Hydrodynamic diameters calculated from the FCS autocorrelation curves in d,e demonstrating co-assembly for PEG-0.3, whilst the control mixture yielded two different sizes. (h) DLS size distributions (intensity) for PEG-0.3 incubated in PBS or 10% (v/v) FBS at 37 °C. (i) Mean sizes from h (mean ± s.e.m., technical triplicates). Box-plots: Center line, the median; box limits, upper and lower quartiles; whiskers, minimum and maximum values.

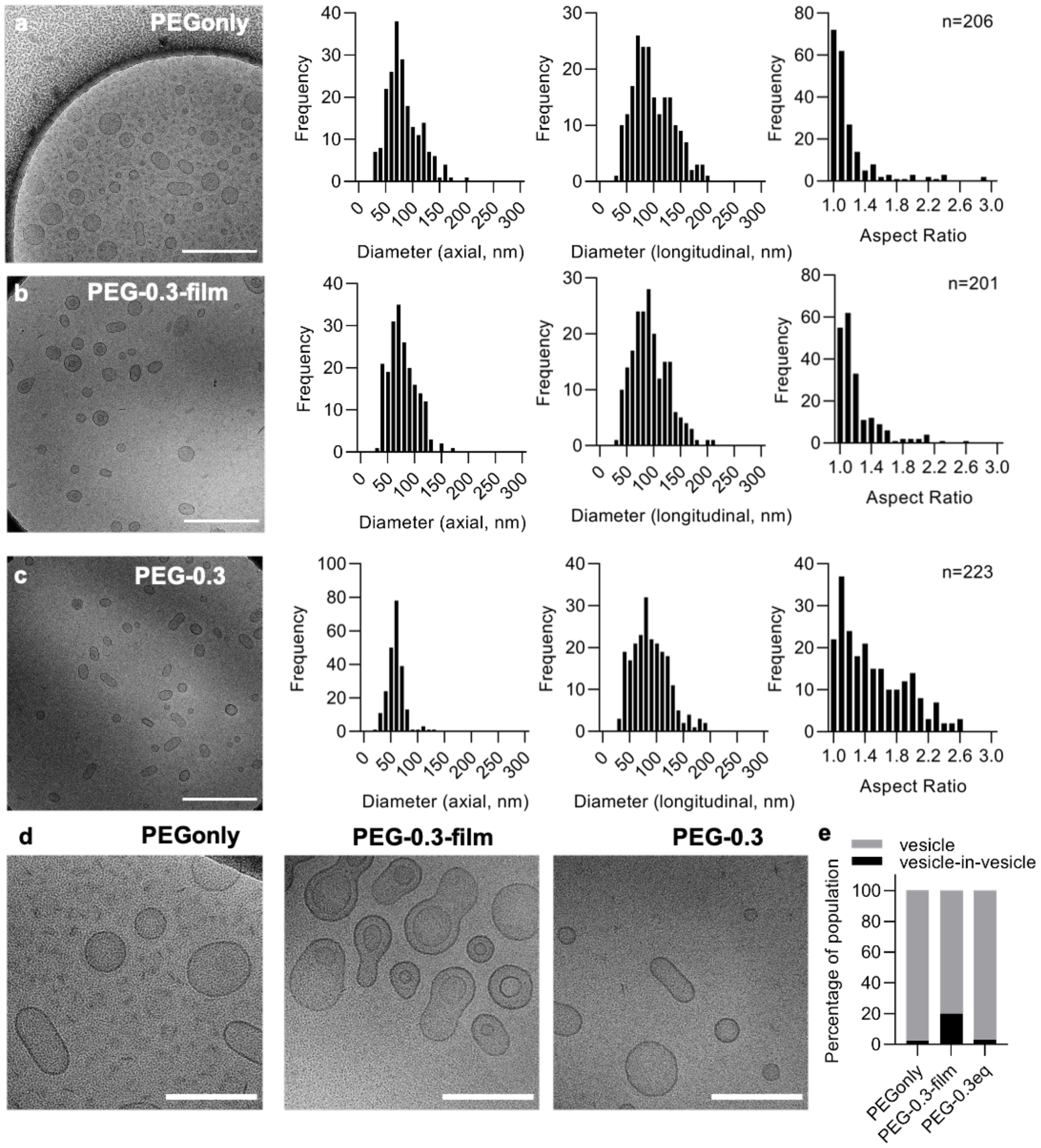

Supplementary Figure 8. Cryo-TEM analysis of PLNs. (a) Representative overview image of PEGonly including histograms for axial and longitudinal axis and corresponding aspect ratio (n = 206 particles, membrane 5.8 ± 0.8 nm). Scale bar, 500 nm. (b) Representative overview image of PEG-0.3-film including histograms for axial and longitudinal axis and corresponding aspect ratio (n = 201 particles, membrane 5.9 ± 1.0 nm). Scale bar, 500 nm. (c) Representative overview image of PEG-0.3 including histograms for axial and longitudinal axis and corresponding aspect ratio (n = 223 particles, membrane 5.8 ± 0.6 nm). Scale bar, 500 nm. (d) Zoomed in images of the three samples. Scale bar, 200 nm. (e) Percentage of vesicle-in-vesicle structures versus unilamellar vesicles (n > 200 particles).

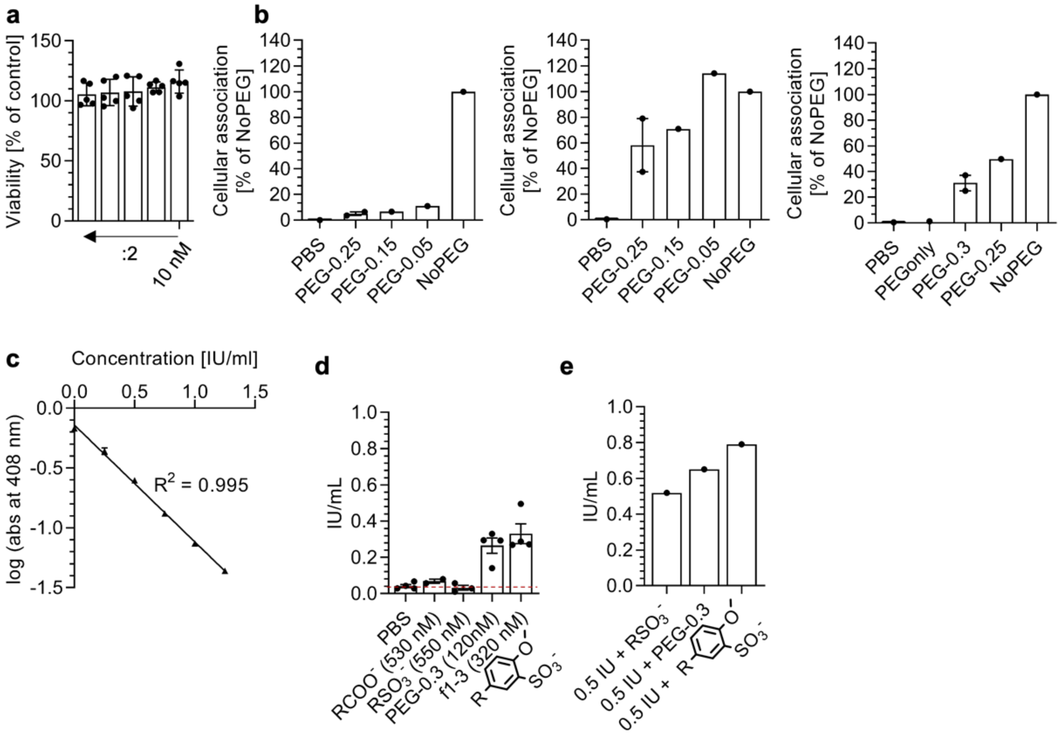

Supplementary Figure 9. Cell viability, cell association and anticoagulation activity evaluation of nanomimics. (a) Cell viability of RAW 264.7 cells after incubation with PEG-0.3 (N = 5 independent experiments with technical triplicates). (b) Cellular association: relative median fluorescence intensity (% of NoPEG control) of RAW 264.7 cells after incubation with DiD-labeled vesicles for 2 h in presence of 10% (v/v) FBS and analysis by flow cytometry (total N = 3 independent experiments plotted separately; each dot represents average of technical duplicates for independent samples). (c) Calibration curve of anticoagulation assay (anti-factor Xa tests) using heparin standards. (d) Interpolated anti-factor Xa activity for various nanomimic samples: AMSA 0.03 ± 0.02 USP/mL (at 720 μg/mL); AMBS 0.33 ± 0.11 USP/mL (at 760 μg/mL); and PLNs 0.26 ± 0.08 USP/mL (at 290 μg/mL). This equals to 0.02%, 0.23%, and 0.48%, respectively, compared to heparin on a weight basis. ($N \geq 2$ independent experiments in duplicates, dotted red line shows PBS background value). (e) Spike controls for d; mixing nanomimics with known amounts of heparin, showing good recovery, hence confirming suitability of the assay with nanoparticles present (N = 1 independent experiment in duplicates).

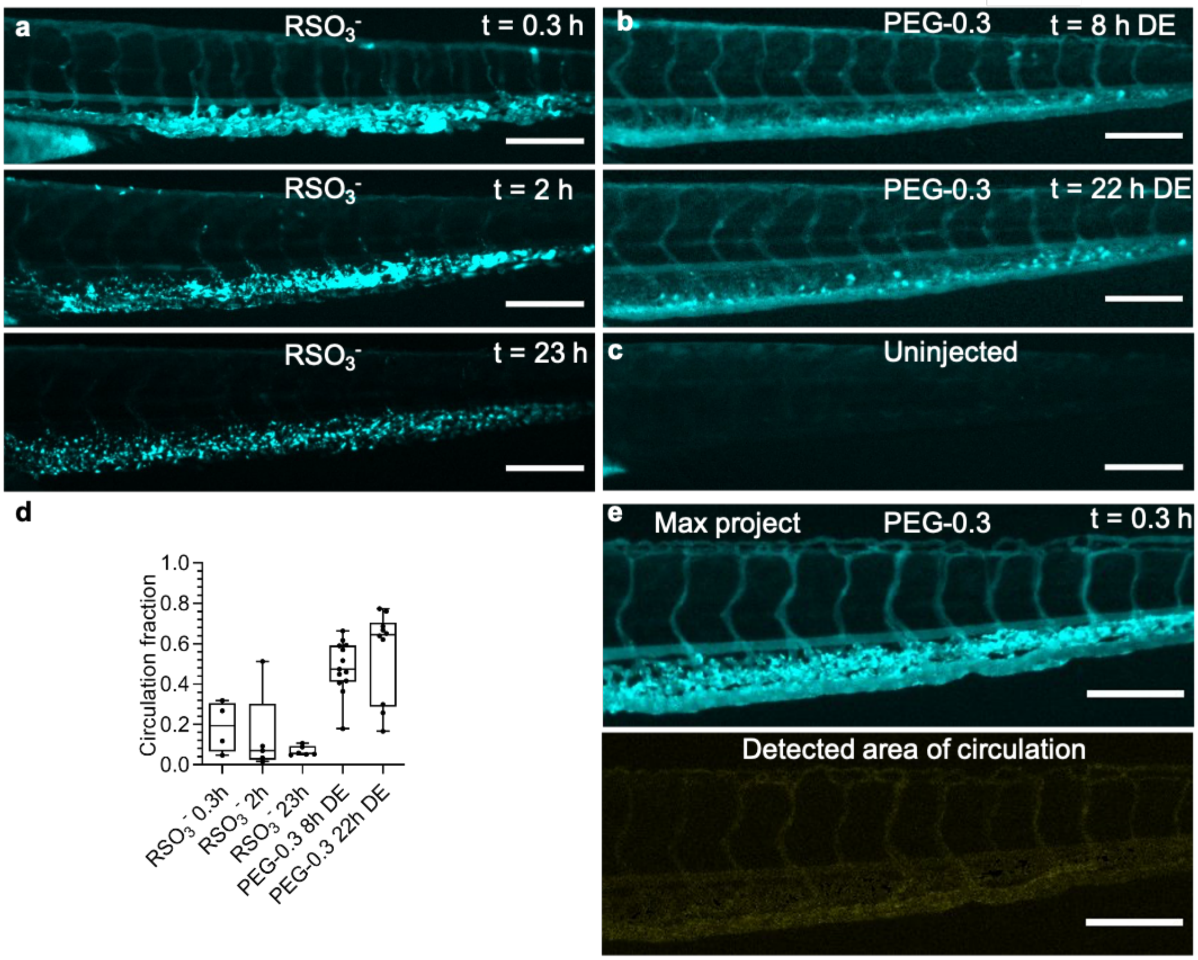

Supplementary Figure 10. Bloodstream circulation of nanomimics in zebrafish embryos. Fluorescence micrograph of zebrafish embryo tail for different samples at different timepoints after nanoparticle injection. (a) AMSA-modified polymer nanoparticles (CF488 labeled), (b) PEG-0.3 (DiO labeled), (c) uninjected control. Note in b for later timepoints (8 and 22 h) exposure time was doubled compared to Figure 2 of main text due to signal decrease (bleaching) over time. (d) Circulation fraction for AMSA nanoparticles and PEG-0.3 at later time points. (e) Example of a max projection of total fluorescence (top) and detected area of circulation (bottom, mainly showing vasculature) from example video used for circulation analysis. Scale bars, 200 μm. Box-plots: Center line, the median; box limits, upper and lower quartiles; whiskers, minimum and maximum values.

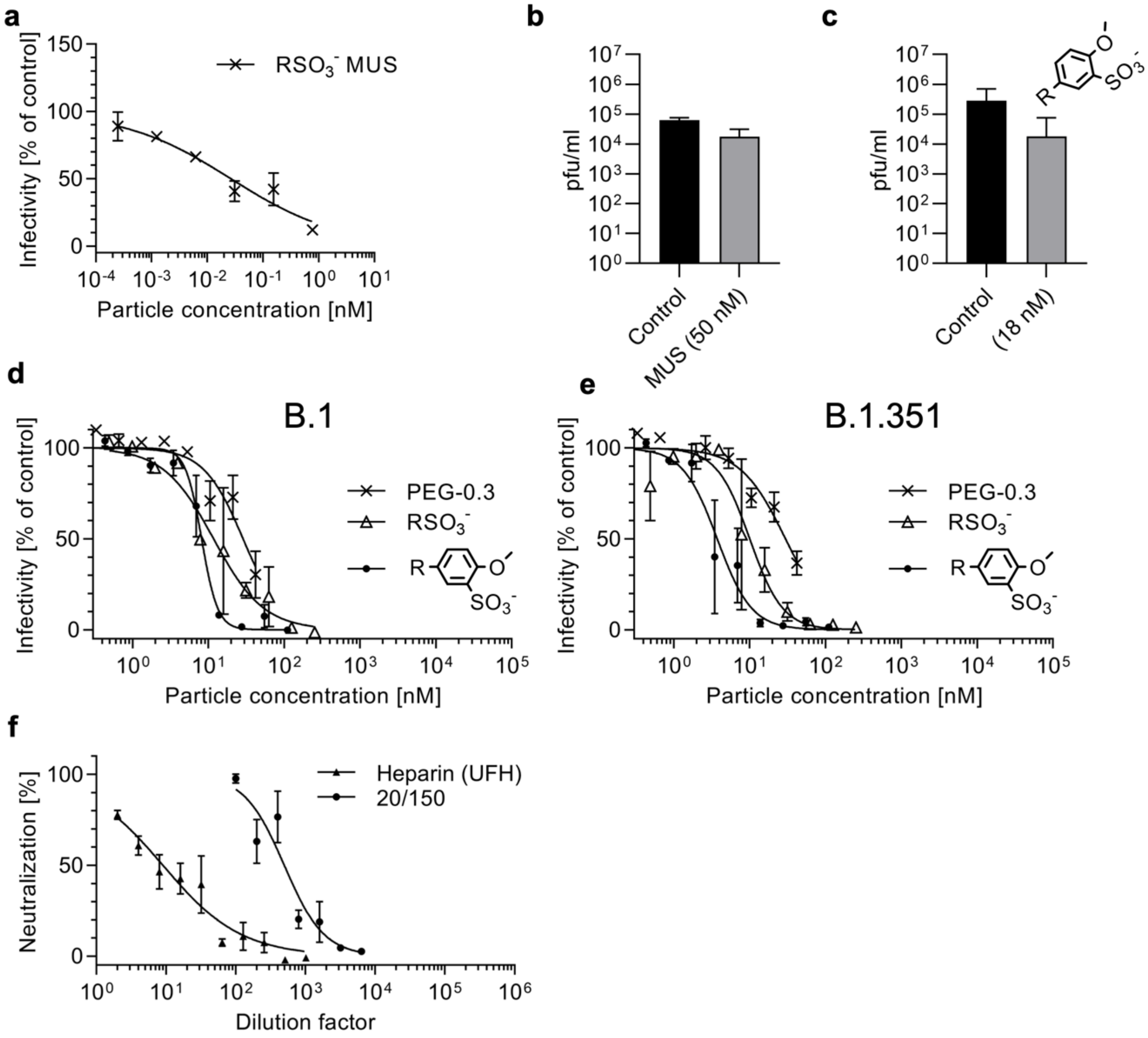

Supplementary Figure 11. Antiviral activity of polymer nanomimics. (a) Dose-response curves for HSV-2 inhibition using MUS-end modified AMSA nanoparticles (mean and range of $N = 1$ independent experiment with technical duplicates). (b) Virucidal test with HSV-2 and MUS-end modified AMSA nanoparticles. (c) Virucidal test with HSV-2 and AMBS nanoparticles. (d,e) Dose-response curves (mean and range of $N = 1$ independent experiment with technical duplicates) using two SARS-CoV-2 variants B.1 and Beta variant B.1.351 and the three main nanoparticles AMSA and AMBS modified polymer nanoparticles and hybrids PEG-0.3. (f) Dose-response curves (mean ± s.e.m., $N \geq 3$ independent experiments with n = 1-2 technical repeats) using SARS-CoV-2 variant B.1, testing heparin (UFH; stocks were 80 mg/mL, lowest dilution 1:2) and WHO Reference Panel (anti-SARS-CoV-2 immunoglobulins, NIBSC code: 20/150).[1] Note that y-axis is % neutralization (different to other plots) in order to compare to 20/150, which is evaluated based on a dilution factor. EC_{50} for UFH: 8.9 mg/mL, which is about 500 μM.

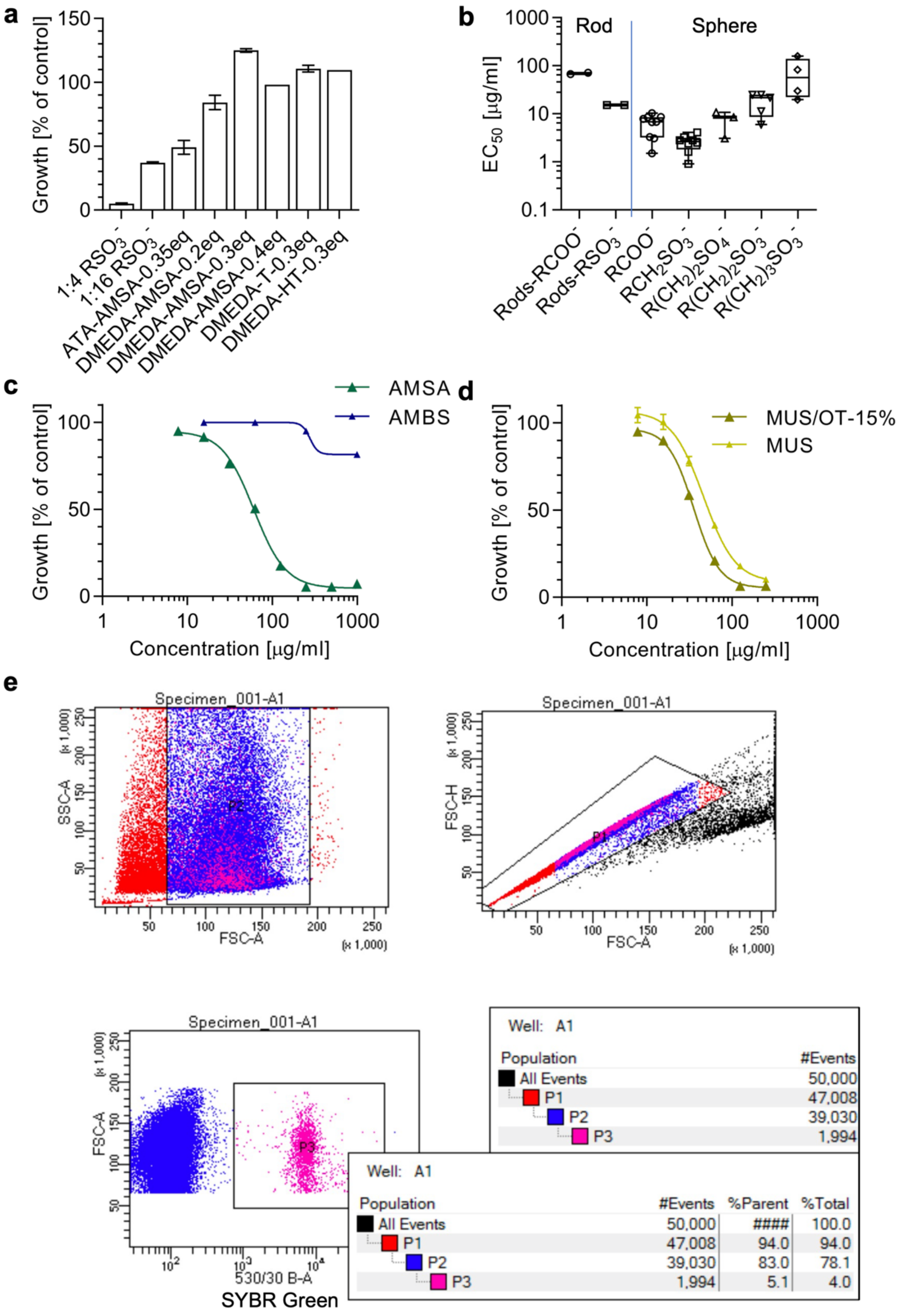

Supplementary Figure 12. ***In vitro*** **parasite inhibition data for alternative nanomimics and building blocks.** (a) Percentage of growth for *P. falciparum* 3D7 when incubated with various anionic and mixed charge polymer nanoparticles (N = 1 independent experiment with technical duplicates). All added stock

particle concentrations were the same, whilst the potent AMSA modified nanoparticles were diluted demonstrating much lower potency when incorporating cationic moieties (see Supplementary Figure 2). (b) EC_{50} values obtained from dose-response curves using *P. falciparum* 3D7 and testing rod shaped vs spherical particles (see Supplementary Figure 1) including increase in spacer length from AMSA, AES, T, to HT ($N \geq 2$ independent experiments in duplicates). (c) Dose-response curves using *P. falciparum* 3D7 and building blocks AMSA and AMBS alone (mean and range of $N = 1$ independent experiment with technical duplicates). (d) Dose-response curves using *P. falciparum* 3D7 and MUS-AuNCs[2] and MUS-OT-15%-AuNCs[2] (mean and range of $N = 1$ independent experiment with technical duplicates). (e) Gating strategy for identifying infected RBCs (SYBR green positive P3) and corresponding parasitemia (P3). Box-plots: Center line, the median; box limits, upper and lower quartiles; whiskers, minimum and maximum values.

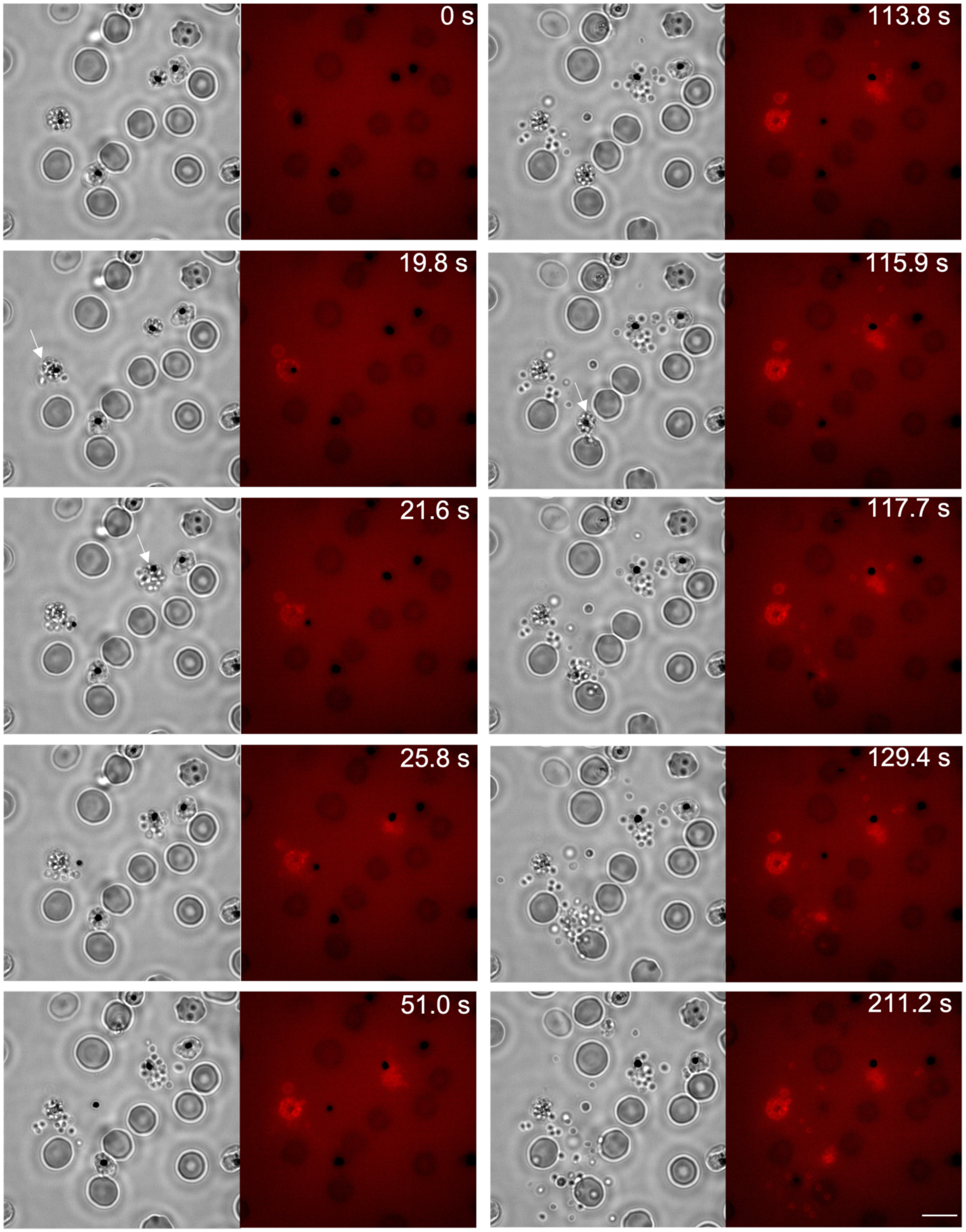

Supplementary Figure 13. Video screen shots of merozoite egress and nanomimic action. Gray phase contrast images show healthy RBCs next to schizont stage *P. falciparum* and AMSA-modified polymer nanomimics (covalently Cy5 labeled, red channel). After schizonts burst, free merozoites and agglutinated merozoites light up in red due to surface accumulation of fluorescent nanoparticles. White arrows indicate

the frame when schizonts burst, elapsed time is given in seconds (See Supplementary Movie S5). Scale bar, 10 μm.

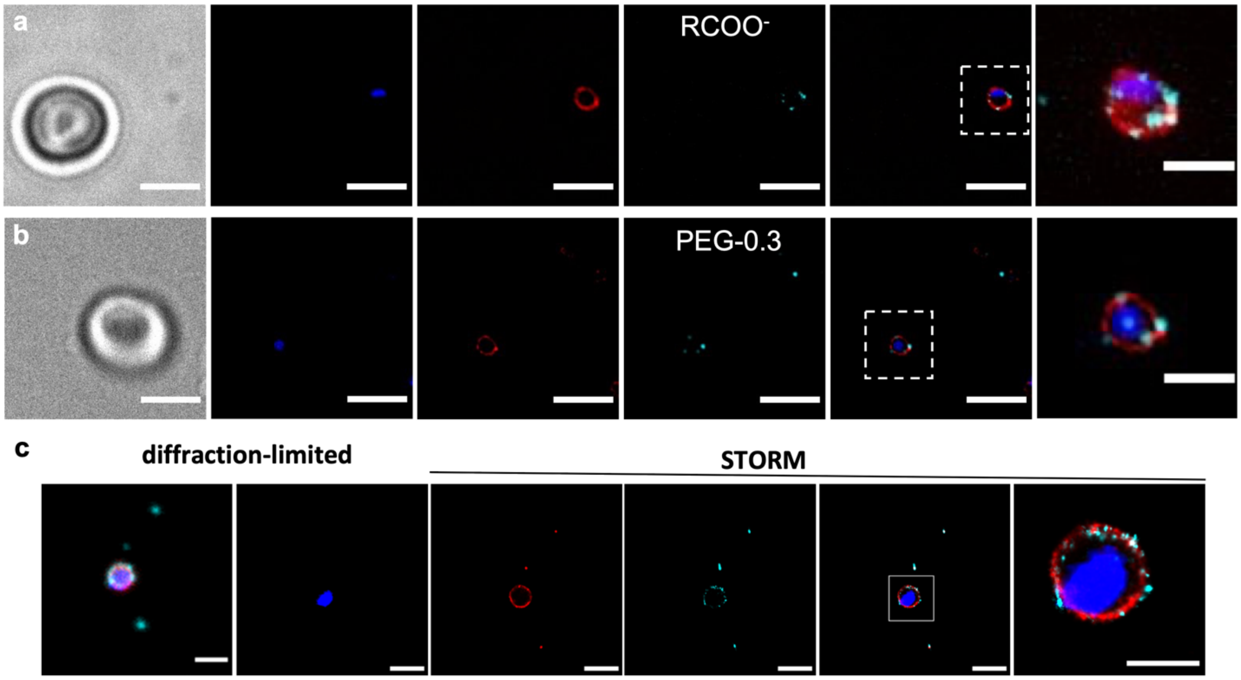

Supplementary Figure 14. Fluorescence images of nanomimic-inhibited merozoites. (a,b) Widefield deconvolution imaging of nanomimic-inhibited (PDLLA-PAA-CF488 and PEG-0.3, respectively, cyan) merozoite (middle slice, nucleus in blue, MSP1 in red) and zoomed z-projection of z-stack. Scale bars, 5 μm and 2 μm (zoom), respectively. (c) Complete panel of figures of main Figure 4f. Diffraction-limited and STORM image of nanomimic- (PDLLA-AMSA-Cy5, cyan) inhibited merozoite (nucleus in blue, MSP1 in red). Three dots appearing in red/cyan channel are fiducial markers used to align images. Zoomed and merged image (right) is shown in main Figure 4f. Scale bars, 2 μm and 1 μm (zoom), respectively.

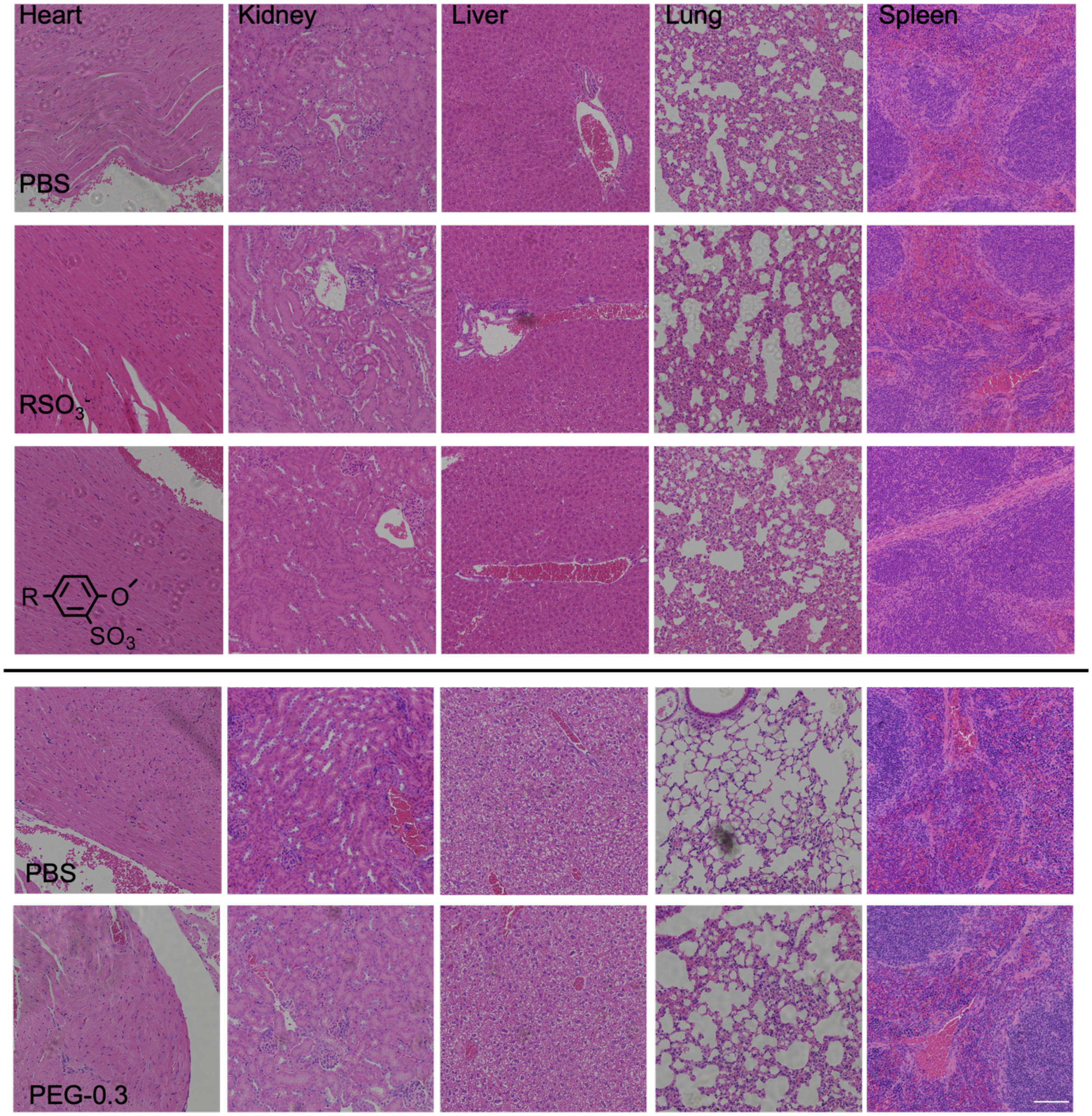

Supplementary Figure 15. H&E-stained histology sections of mouse tissues, x20 objective. BALB/c mice were i.v. injected with particle solutions in PBS at 1.5 mg/kg and compared to a PBS control. Top three are day 4 and bottom two are day 9. Tissues were fixed, embedded in paraffin, sectioned, and stained with hematoxylin and eosin (H&E). Scale bar, 100 μm.

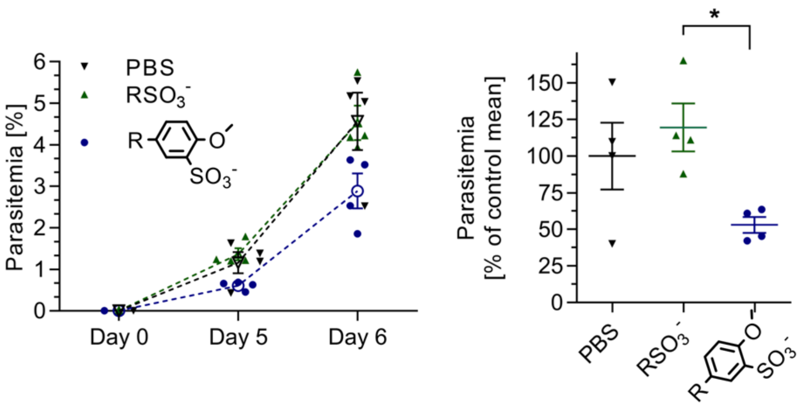

Supplementary Figure 16. ***In vivo*** **parasite inhibition data for AMSA- and AMBS-based nanomimics.** Conditions were: 1 x 10^5 *P. berghei*-infected RBCs (schizonts) and 1.5 mg/kg treatment on day 0. Parasitemia followed over time (dotted lines and open symbols represent mean ± s.d.) and corresponding plot of % inhibited *vs* PBS control at day 5 when parasitemia reached > 1% for PBS group (n = 4 mice per group, pooled from two independent experiments, one-way ANOVA with Tukey's multiple comparisons test, *P < 0.05).

**Parameter Name**	**Fitted Value**	**Value Error**	**Fixed / Fitted**
scale	0,0060321	0,00011709	Fitted
background (cm^{-1})	-0,00045782	0,00022192	Fitted
radius_equat_core (Å)	861,03	10,318	Fitted
x_core	1,1908	0,060564	Fitted
thick_shell (Å)	11,778	1,2499	Fitted
x_polar_shell	5,1052	0,61235	Fitted
sld_core ($x10^{-6}$ /$Å^2$)	5		Fixed
sld_shell ($x10^{-6}$ /$Å^2$)	1		Fixed
sld_solvent ($x10^{-6}$ /$Å^2$)	6,3		Fixed

Table S1. SANS fitting parameters for Core Shell Ellipsoid model for PEGonly.

Parameter Name	Fitted Value	Value Error	Fixed / Fitted
scale	0,0075966	0,000161	Fitted
background (cm^{-1})	0,0028414	0,00022205	Fitted
sld_core ($x10^{-6}$ /$Å^2$)	5,5397	0,016037	Fitted
radius (Å)	962,66	0,73197	Fitted
sld_solvent ($x10^{-6}$ /$Å^2$)	6,4		Fixed
n	2		Fixed
sld1 ($x10^{-6}$/$Å^2$)	4,5986		Fixed
thickness1 (Å)	75,919	0,62001	Fitted
sld2 ($x10^{-6}$/$Å^2$)	0,85356		Fixed
thickness2 (Å)	40,591	0,76318	Fitted

Table S2. SANS fitting parameters for Core Multi Shell model for PEG-0.3-film.

Supplementary Movies

Movie S1.

Example movie of NoPEG in zebrafish embryo, 0.3 h post injection. See screenshot of this movie and analysis in Figure 2, G to H. Scale bar, 200 μm.

Movie S2.

Example movie of PEG-0.3 in zebrafish embryo, 0.3 h post injection. See screenshot of this movie and analysis in Figure 2, G to H. Scale bar, 200 μm.

Movie S3.

Example movie of PEG-0.3 in zebrafish embryo, 1 h post injection. See screenshot of this movie and analysis in Figure 2, G to H. Scale bar, 200 μm.

Movie S4.

Example movie of PEG-0.3 in zebrafish embryo, 5 h post injection. See screenshot of this movie and analysis in Figure 2, G to H. Scale bar, 200 μm.

Movie S5.

Video of merozoite egress and nanomimic action. See screenshots of this movie and information in Supplementary Figure 13. Scale bar, 10 μm.

Supplementary References

(1) WHO International Laboratory for Biological Standards. *WHO Reference Panel First WHO International Reference Panel for Anti-SARS-CoV-2 Immunoglubulin NIBSC Code: 20/268 Instructions for Use (Version 3.0, Dated 17/12/2020)*; 2020.

(2) Cagno, V.; Andreozzi, P.; D'Alicarnasso, M.; Silva, P. J.; Mueller, M.; Galloux, M.; Goffic, R. Le; Jones, S. T.; Vallino, M.; Hodek, J.; Weber, J.; Sen, S.; Janecek, E. R.; Bekdemir, A.; Sanavio, B.; Martinelli, C.; Donalisio, M.; Welti, M. A. R.; Eleouet, J. F.; Han, Y.; Kaiser, L.; Vukovic, L.; Tapparel, C.; Král, P.; Krol, S.; Lembo, D.; Stellacci, F. Broad-Spectrum Non-Toxic Antiviral Nanoparticles with a Virucidal Inhibition Mechanism. *Nature Materials* **2018**, *17* (2), 195–203.
